# Association of the CUN-BAE index with glycemic outcomes in individuals with impaired fasting glucose: a retrospective multicenter Chinese cohort study

**DOI:** 10.3389/fnut.2026.1813094

**Published:** 2026-09-10

**Authors:** Duo Yang, Renzhe Lin, Sen Li, Shujun Ye, Zitian Luo, Huankai Zhang, Si Wu, Longsheng Zhang

**Affiliations:** 1 Department of Anesthesiology, Jieyang People's Hospital, Jieyang, Guangdong, China; 2 First Clinical Medical College, Guangdong Medical University, Zhanjiang, Guangdong, China

**Keywords:** body fat percentage, body mass index, CUN-BAE index, diabetes, endocrinology, impaired fasting glucose, risk markers

## Abstract

**Background:**

Diabetes mellitus (DM) poses a significant global public health challenge, with impaired fasting glucose (IFG) representing a critical prediabetic state. The Clínica Universidad de Navarra-Body Adiposity Estimator (CUN-BAE) index, a body fat estimator integrating body mass index (BMI), age, and gender, has shown promise in assessing cardiometabolic risk. However, its longitudinal association with bidirectional glycemic transitions, specifically reversion to normoglycemia and progression to DM, in individuals with IFG remains unclear. This study aimed to evaluate the association between baseline CUN-BAE index and glycemic outcomes in a Chinese IFG population.

**Methods:**

This retrospective multicenter cohort study included 26,247 adults with baseline IFG from a health screening program. The CUN-BAE index was calculated using a standardized formula. Cox proportional hazards regression, restricted cubic spline models, and threshold effect analyses were employed to assess associations between the CUN-BAE index and glycemic outcomes, with adjustments for confounders. Subgroup and sensitivity analyses were conducted to verify robustness.

**Results:**

Higher CUN-BAE index levels were independently associated with a reduced probability of reversion to normoglycemia (adjusted HR 0.99 per unit increase; 95% CI 0.98–0.99) and an increased risk of DM progression (adjusted HR 1.04; 95% CI 1.03–1.04). Nonlinear relationships were identified, with threshold effects at CUN-BAE index = 24.53 for reversion and CUN-BAE index = 22.51 for progression. The CUN-BAE index quartile analysis demonstrated a graded association, with the highest CUN-BAE index quartile showing a significantly elevated risk of diabetes and a reduced rate of reversion to normoglycemia.

**Conclusion:**

Higher CUN-BAE index levels are strongly and independently associated with adverse glycemic transitions in IFG individuals, with higher values indicating poorer outcomes. Its nonlinear associations and threshold effects support its utility for risk stratification and personalized intervention strategies in prediabetes management.

## Background

Diabetes mellitus (DM) poses a formidable global public health challenge, with an estimated 537 million affected adults worldwide, a number projected to approach 900 million by 2050 (1, 2). China currently bears the highest disease burden, accounting for approximately 22% (over 118 million) of the global diabetic population (3, 4). Consequently, early intervention in prediabetes, particularly impaired fasting glucose (IFG), has become a critical clinical priority. In China, prediabetes affects up to 35.2% of the population, presenting a massive challenge for precise DM prevention (3). The IFG state is characterized by insulin resistance and *β*-cell dysfunction, carrying an annual 5–10% risk of progression to DM, alongside an elevated long-term risk of cardiovascular, microvascular, and malignant complications (5). However, the IFG stage also represents a crucial therapeutic window. Effective lifestyle interventions can significantly reduce the risk of DM progression and facilitate reversion to normoglycemia (6, 7). Such reversion or delayed progression effectively mitigates the long-term risk of cardiometabolic and microvascular complications (5, 8). Therefore, accurately identifying individuals within this heterogeneous IFG population who are prone to DM progression versus normoglycemic reversion is critical for tailoring precise early-intervention strategies (9).

Obesity is a central driver in the progression from IFG to DM, closely linked to insulin resistance and a chronic low-grade inflammatory state (10). While body mass index (BMI) has become the most widely used screening tool for assessing obesity, it cannot distinguish between fat mass and fat-free mass, leading to a notable “misclassification” phenomenon in metabolic risk assessment (11–13). Consequently, there is a pressing clinical need for alternative indicators that can more accurately assess body fat content to improve risk stratification among individuals with IFG.

To overcome the limitations of traditional metrics, the Clínica Universidad de Navarra-Body Adiposity Estimator (CUN-BAE) index, which integrates BMI, age, and gender, has emerged as a valuable tool for estimating body fat percentage (14–16). Existing literature has highlighted its significant association with various cardiometabolic diseases (17–19). Specifically regarding glycemic outcomes, studies by Peng et al. (20), Geng et al. (21), and Dominguez et al. (22) have demonstrated a robust association between higher CUN-BAE index levels and an increased incidence of DM.

However, these prior works have predominantly focused on the association between CUN-BAE and diabetes incidence in the general population. To date, no study has yet evaluated the CUN-BAE index specifically within the high-risk IFG population nor examined its association with bidirectional glycemic outcomes—namely, reversion to normoglycemia and progression to DM—simultaneously. This gap constitutes the primary scientific rationale for the present study. Based on a large-scale, multicenter Chinese retrospective cohort, this study aims to systematically investigate the association of the baseline CUN-BAE index with bidirectional glycemic outcomes in individuals with IFG and to explore potential nonlinear relationships and threshold effects.

## Method

### Data source

The original dataset used in this study was sourced from the Dryad data repository, a digital resource supporting open access that integrates a wide range of publicly available research data across multiple disciplines following the principles of open science. The dataset referenced in this study is associated with a large cohort study focused on Chinese adults, published in *BMJ Open* in 2018, titled “Association of body mass index and age with incident diabetes in Chinese adults: a population-based cohort study” (23). Researchers can access the complete data resource via its Digital Object Identifier link.^1^ This dataset contains longitudinal clinical follow-up data from participants in a health screening program between 2010 and 2016. In accordance with the relevant policies of the Dryad platform, data hosted on the platform may be used for secondary analyses to explore new scientific questions. Since the original study had already obtained ethical review approval from the Chinese Rich Healthcare Group, this secondary analysis of the same dataset does not require renewed ethical approval or re-consent from the participants. Furthermore, all procedures in this study strictly adhered to the ethical standards outlined in the Declaration of Helsinki and fully complied with national relevant laws, regulations, and institutional governance guidelines.

### Study population

**Figure 1:**
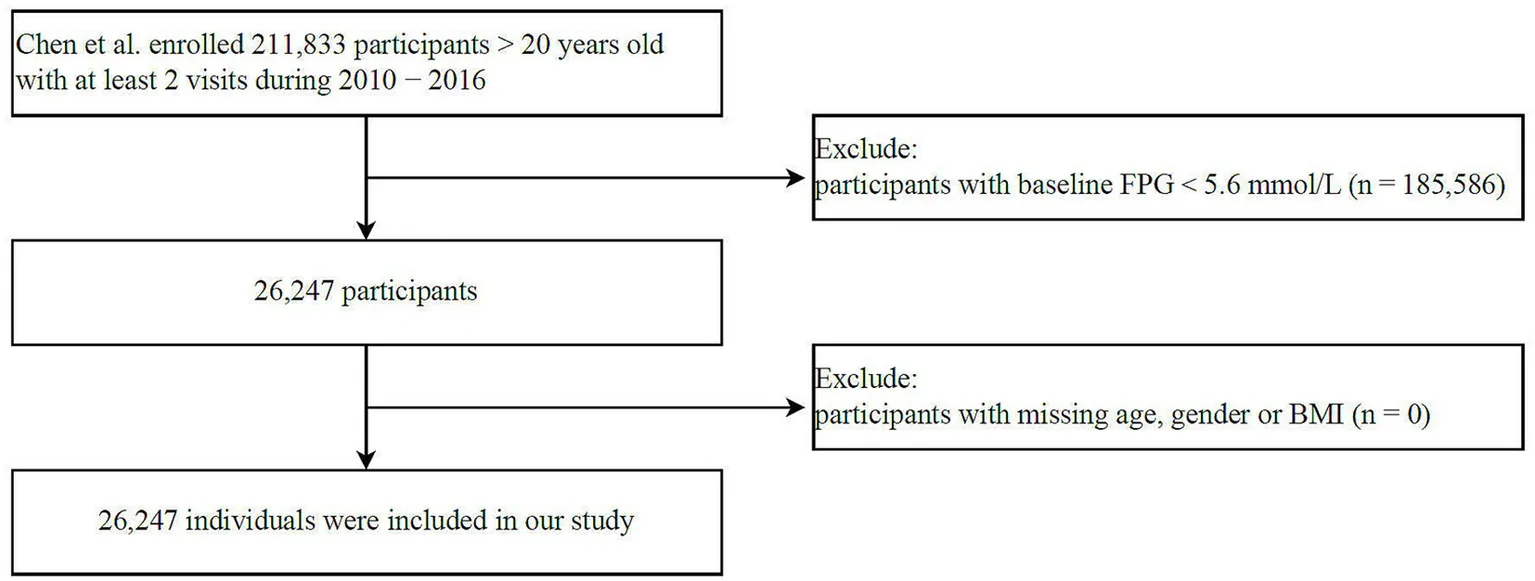
Flowchart of the study.

This secondary analysis was conducted based on a cohort previously established by Chen et al. in a mainland Chinese population. The original cohort initially included 685,277 participants. According to predefined inclusion and exclusion criteria, 211,833 individuals were ultimately enrolled to form the preliminary study population (23). The present analysis focused specifically on individuals with IFG at baseline, aiming to evaluate the longitudinal association of the baseline CUN-BAE index with transitions in glycemic status. Based on the predetermined exclusion criteria, we excluded individuals with baseline fasting blood glucose below 5.6 mmol/L, as well as those with missing data on age, gender, or BMI. Consequently, a total of 26,247 participants were included in the final analysis. The detailed flowchart of participant selection is shown in Figure 1.

### Definitions of the exposure variable

The primary exposure variable in this study was the CUN-BAE index, which was treated as a continuous variable. It was estimated using the formula proposed in the literature (14). Specifically, the CUN-BAE index, used to assess the percentage of body fat, was calculated as follows:


$$\begin{array}{l} \mathrm{CUN}-\mathrm{BAE}\;\text{index}=-44.988+(0.503^{*}\;\mathrm{age})+(10.689^{*}\;\text{gender})+\\ (3.172^{*}\;\mathrm{BMI})-(0.026^{*}\;{\mathrm{BMI}}^{2})+\\ (0.181^{*}\;{\mathrm{BMI}}^{*}\;\text{gender})-(0.02^{*}\;{\mathrm{BMI}}^{*}\;\mathrm{age})-\\ (0.005^{*}\;{\mathrm{BMI}}^{2*}\;\text{gender})+(0.00021^{*}\;{\mathrm{BMI}}^{2*}\;\mathrm{age}) \end{array}$$

Where male is coded as 0, female as 1, and age is expressed in years.

### Definitions of the outcome variable

The primary endpoints of this study were transitions in glycemic status, specifically encompassing the following two outcomes: (1) reversion to normoglycemia and (2) progression to DM. The classification of these glycemic statuses was based on the diagnostic criteria recommended by the American Diabetes Association (ADA). Specifically, an individual was classified as having normoglycemia if their fasting plasma glucose (FPG) concentration at the last health examination was below 5.6 mmol/L and they had no self-reported history of DM. A DM event was defined as an FPG level of ≥ 7.0 mmol/L at the final examination or a self-reported physician diagnosis of DM. Furthermore, the follow-up duration was defined as the entire observation period from the date of the initial health screening to the date of the final health examination.

To enhance international generalizability, a sensitivity analysis redefining baseline IFG according to the World Health Organization (WHO) criteria (FPG 6.1–6.9 mmol/L) was also performed.

### Other covariates

The covariates considered in this study encompassed the following categories: (1) demographic characteristics, including age and gender; (2) anthropometric measurements, involving height, weight, and blood pressure (BP); (3) blood biochemical parameters, specifically fasting blood glucose, total cholesterol (TC), triglycerides (TG), low-density lipoprotein cholesterol (LDL-C), high-density lipoprotein cholesterol (HDL-C), alanine aminotransferase (ALT), aspartate aminotransferase (AST), blood urea nitrogen (BUN), and serum creatinine (Scr); (4) behavioral factors, such as smoking and drinking status; and (5) family history of DM. Information on demographic characteristics, lifestyle behaviors, and family disease history was collected via structured questionnaires. Anthropometric measurements, including height, weight, and BP, were obtained by uniformly trained staff following standardized protocols. BMI was calculated as weight in kilograms divided by the square of height in meters. Based on the categorical definitions in the original database, smoking and drinking status were classified as “current,” “former,” or “never.” Venous blood samples were collected by professional personnel from all participants after a fast of at least 10 h. All biochemical parameters were measured using a Beckman Coulter AU5800 fully automated biochemical analyzer.

### Missing data processing

Missing data existed for some variables in the dataset used in this study. To address this, standard strategies for handling missing data in observational epidemiological research were employed. Among the variables included in the analysis, a total of 12 variables contained missing values, comprising 10 continuous variables and 2 categorical variables. Specifically, variables with missingness exceeding 15% included AST (56.578%), HDL-C (40.664%), LDL-C (38.248%), and smoking and drinking status (66.301%). The specific proportion of missing data for each variable is listed in Supplementary Table 1. Regarding missing records for the categorical variables of smoking and drinking status, this study employed a strategy of uniformly classifying them as “not recorded.” For the multiple imputation procedure, we operated under the assumption that the data were missing at random. To reduce potential bias introduced by missing data, multiple imputation was performed for covariates using the MICE package in the R statistical environment (24). The Multiple Imputation by Chained Equations method was applied, generating 5 complete datasets after 5 iterations.

To assess the reliability of the multiple imputation method, the main regression analyses reported in the main text are based on the pooled datasets after imputation. To comprehensively verify the stability of the conclusions, analyses using only the original complete observation data (pre-imputation) and data obtained after deleting participants with incomplete covariate information are also provided in Supplementary materials. Furthermore, to explicitly address potential concerns regarding the reliability of imputed estimates, we conducted an additional sensitivity analysis. In this model, variables with a missing rate > 15% were not included as adjustment covariates. Instead, the model was re-constructed to adjust only for variables with complete or near-complete data (SBP, DBP, FPG, TC, TG, ALT, BUN, and Scr). The alignment of outcomes across all these analytical frameworks indicates that the main findings of this study are not sensitive to the method of handling missing data, demonstrating excellent robustness of the results.

### Statistical method

**Figure 2:**
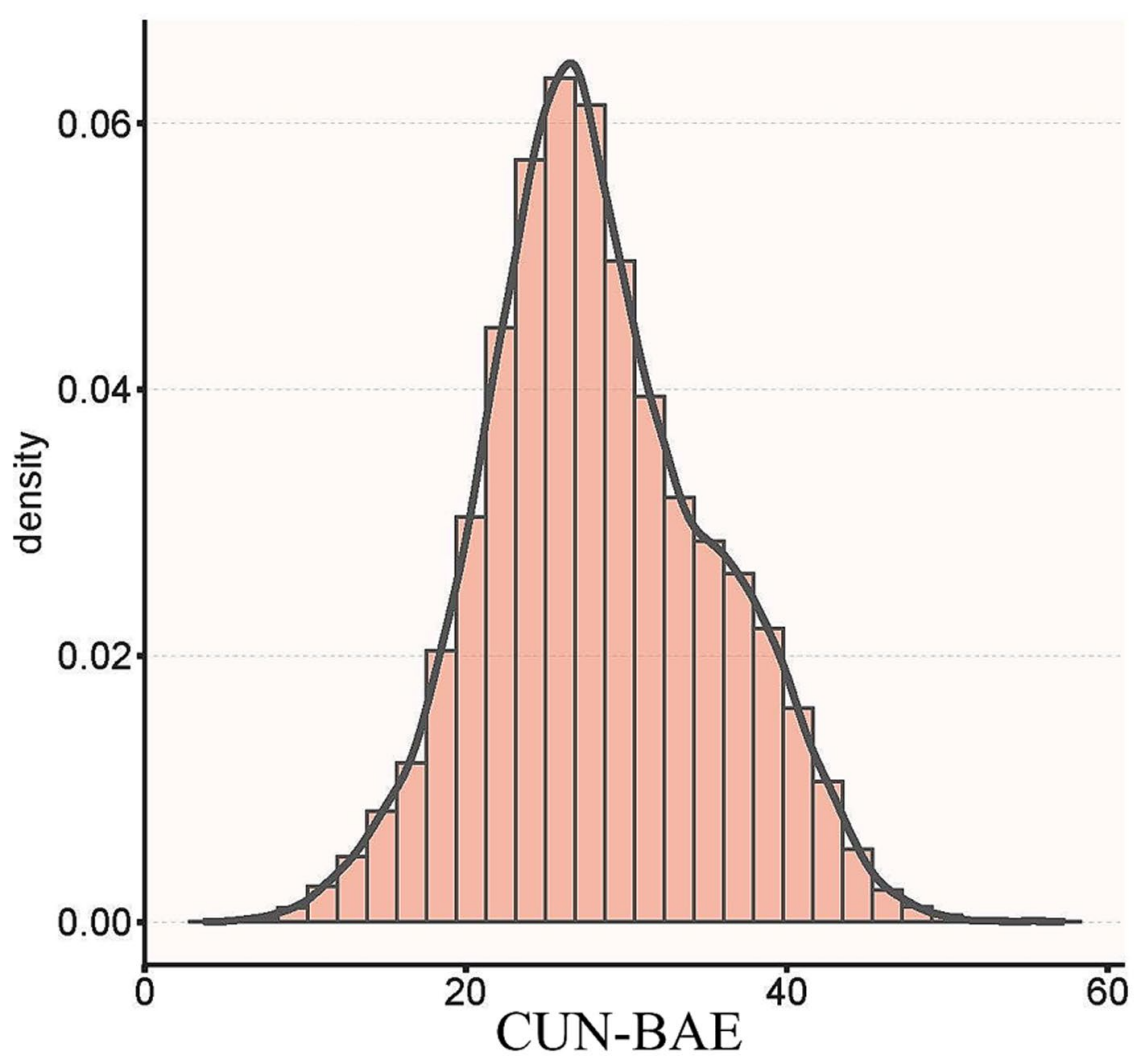
The distribution of the CUN-BAE index.

The distribution of CUN-BAE index across all participants is presented in Figure 2. Based on quartiles of CUN-BAE index, all participants were divided into four groups: Q1 (≤ 23.508), Q2 (≤ 27.502), Q3 (≤ 32.868), and Q4 (≤ 57.297). The normality of continuous variables was assessed using the Kolmogorov–Smirnov test. Variables conforming to a normal distribution are presented as mean ± standard deviation, while those with a non-normal distribution are described as median (interquartile range) [M (IQR)]. Categorical variables are expressed as frequency (percentage) [n (%)]. For inter-group comparisons, one-way analysis of variance (ANOVA) was used for normally distributed continuous variables with homogeneity of variance; otherwise, the Kruskal-Wallis H test was applied. Categorical variables were compared using the chi-square test.

Transitions in glycemic status were visualized using Kaplan–Meier (K-M) curves. Differences in transition probabilities among CUN-BAE index quartile groups were statistically assessed using the log-rank test. Multivariable Cox proportional hazards regression models were employed to evaluate the association between the estimated CUN-BAE index and transitions in glycemic status. After adjusting for potential confounding factors, hazard ratios (HRs) and 95% confidence intervals (CIs) were estimated. Specifically, two progressively adjusted models were constructed: Model I was adjusted for baseline FPG, systolic blood pressure (SBP), diastolic blood pressure (DBP), and family history of DM; Model II additionally incorporated the following baseline covariates: ALT, AST, LDL-C, HDL-C, TC, TG, BUN, Scr, smoking status, and drinking status. To verify the robustness of the relationship between CUN-BAE index and glycemic status transitions from multiple perspectives, a multivariable logistic regression model was constructed in parallel. This model estimated odds ratios (ORs) and 95% CIs, adjusting for the same set of variables as specified in the Cox models, to examine the consistency of the associations under different methodological frameworks. Additionally, given the mutually exclusive nature of the bidirectional glycemic transitions (i.e., reversion to normoglycemia and progression to DM act as competing events for each other), a competing risk regression using the Fine-Gray proportional subdistribution hazards model was performed as an important sensitivity analysis. Concordant statistical conclusions from both regression models would significantly strengthen the credibility of the findings.

To further investigate potential nonlinear associations between CUN-BAE index and glycemic outcomes, a restricted cubic spline (RCS) was used to fit a smooth function curve. If a nonlinear trend was suggested, a piecewise regression model was applied to identify potential threshold effects or inflection points. Furthermore, to evaluate the stability of the relationship between CUN-BAE index and glycemic status transitions across different population characteristics, stratified Cox regression analyses were performed. Stratification was based on gender, age, BMI, SBP, DBP, smoking status, and drinking status. Analyses within each subgroup were uniformly adjusted for a series of covariates—including baseline FPG, SBP, DBP, ALT, AST, LDL-C, HDL-C, TC, TG, BUN, Scr, smoking status, drinking status, and family history of DM to control for residual confounding, with the stratification variable itself excluded from adjustment within its respective stratum.

### Statistical software

All results are reported in accordance with the strengthening the reporting of observational studies in epidemiology (STROBE) statement. Statistical analyses were performed in the R environment (version 4.2.2; https://www.R-project.org/) and the Free Statistics Analysis Platform (version 2.1.1; https://www.clinicalscientists.cn/freestatistics). A two-tailed *p*-value of less than 0.05 was considered statistically significant. As the present study constitutes a secondary analysis of pre-existing data, no formal *a priori* sample size calculation was performed.

## Results

### Baseline characteristics of the study population

This study ultimately included 26,247 individuals with IFG at baseline. The population was stratified into quartiles (Q1 to Q4) based on baseline CUN-BAE index values. All demographic and clinical characteristics differed significantly among the quartile groups (all *p* < 0.001). As shown in Table 1, as the CUN-BAE index quartile increased (from Q1 to Q4), the study population showed trends of progressively higher age, a greater proportion of females, and increased BMI. Levels of SBP, DBP, baseline FPG, TC, and LDL-C, as well as the proportion with a positive family history of DM, also showed a gradual upward trend with increasing CUN-BAE index. In contrast, height and Scr levels decreased progressively with higher CUN-BAE index quartiles (all *p* < 0.001). The median values of body weight, ALT, AST, BUN, and TG, as well as the distributions of smoking and drinking status, also differed significantly among the quartile groups (all *p* < 0.001).

**Table 1:** Baseline demographic and clinical characteristics of the study cohort, stratified by CUN-BAE index quartiles.

Variables	Total (*n* = 26,247)	CUN-BAE index				
		Q1 (*n* = 6,560)	Q2 (*n* = 6,560)	Q3 (*n* = 6,565)	Q4 (*n* = 6,562)	*p*-value
Age (years), Mean ± SD	49.11 ± 13.82	41.58 ± 11.77	49.10 ± 13.06	50.85 ± 13.87	54.92 ± 12.99	< 0.001
Gender, *n* (%)						< 0.001
Male	17,424 (66.38)	6,354 (96.86)	5,880 (89.63)	4,488 (68.36)	702 (10.70)	
Female	8,823 (33.62)	206 (3.14)	680 (10.37)	2077 (31.64)	5,860 (89.30)	
BMI (kg/m^2^), Mean ± SD	24.82 ± 3.37	22.19 ± 2.02	24.78 ± 2.27	26.00 ± 3.53	26.29 ± 3.65	< 0.001
Height (cm), Mean ± SD	166.70 ± 8.34	171.20 ± 6.58	169.46 ± 6.73	166.76 ± 7.72	159.37 ± 6.96	< 0.001
Body weight (kg), mean ± SD	69.24 ± 12.24	65.22 ± 8.27	71.49 ± 10.03	73.02 ± 14.62	67.21 ± 13.29	< 0.001
CUN-BAE index, mean ± SD	28.24 ± 7.07	19.71 ± 3.19	25.56 ± 1.15	29.91 ± 1.53	37.78 ± 3.51	< 0.001
SBP (mmHg), mean ± SD	127.22 ± 17.59	123.33 ± 15.15	126.85 ± 17.04	128.29 ± 18.27	130.41 ± 18.91	< 0.001
DBP (mmHg), mean ± SD	78.42 ± 11.15	76.56 ± 10.01	79.38 ± 11.05	79.43 ± 11.80	78.30 ± 11.44	< 0.001
Baseline FPG (mmol/L), Mean ± SD	5.95 ± 0.33	5.89 ± 0.29	5.96 ± 0.33	5.98 ± 0.34	5.98 ± 0.34	< 0.001
TC (mmol/L), mean ± SD	4.98 ± 0.96	4.75 ± 0.90	4.98 ± 0.94	4.98 ± 0.96	5.19 ± 0.98	< 0.001
TG (mmol/L), median (IQR)	1.42 (0.97, 2.13)	1.20 (0.84, 1.77)	1.54 (1.02, 2.30)	1.51 (1.00, 2.30)	1.46 (1.03, 2.11)	< 0.001
HDL-C (mmol/L), mean ± SD	1.33 ± 0.30	1.34 ± 0.30	1.29 ± 0.28	1.31 ± 0.30	1.37 ± 0.30	< 0.001
LDL-C (mmol/L), mean ± SD	2.88 ± 0.72	2.75 ± 0.68	2.87 ± 0.71	2.87 ± 0.71	3.04 ± 0.75	< 0.001
ALT (U/L), median (IQR)	22.00 (15.40, 33.10)	21.00 (15.20, 31.00)	24.50 (17.40, 36.20)	23.90 (15.60, 37.00)	19.50 (14.20, 29.00)	< 0.001
AST (U/L), median (IQR)	24.00 (20.00, 29.90)	23.05 (19.50, 28.00)	24.40 (20.50, 30.00)	25.00 (20.20, 31.00)	23.90 (20.00, 29.00)	< 0.001
BUN (mmol/L), mean ± SD	4.99 ± 1.25	4.99 ± 1.21	5.08 ± 1.25	5.01 ± 1.27	4.88 ± 1.25	< 0.001
Scr (μmol/L), mean ± SD	72.76 ± 16.00	78.63 ± 13.15	77.45 ± 14.04	73.37 ± 16.73	61.60 ± 13.87	< 0.001
Smoking status, *n* (%)						< 0.001
Current smoker	2,261 (8.61)	742 (11.31)	802 (12.23)	634 (9.66)	83 (1.26)	
Ever smoker	418 (1.59)	166 (2.53)	144 (2.20)	92 (1.40)	16 (0.24)	
Never smoker	6,166 (23.49)	1727 (26.33)	1,438 (21.92)	1,433 (21.83)	1,568 (23.90)	
Not recorded	17,402 (66.30)	3,925 (59.83)	4,176 (63.66)	4,406 (67.11)	4,895 (74.60)	
Drinking status, *n* (%)						< 0.001
Current drinker	359 (1.37)	88 (1.34)	133 (2.03)	127 (1.93)	11 (0.17)	
Ever drinker	1,541 (5.87)	581 (8.86)	528 (8.05)	353 (5.38)	79 (1.20)	
Never drinker	6,945 (26.46)	1966 (29.97)	1723 (26.27)	1,679 (25.58)	1,577 (24.03)	
Not recorded	17,402 (66.30)	3,925 (59.83)	4,176 (63.66)	4,406 (67.11)	4,895 (74.6)	
Family history of DM, *n* (%)						< 0.001
No	25,605 (97.55)	6,439 (98.16)	6,435 (98.09)	6,422 (97.82)	6,309 (96.14)	
Yes	642 (2.45)	121 (1.84)	125 (1.91)	143 (2.18)	253 (3.86)	
Outcomes, *n* (%)						< 0.001
Normoglycemia	11,909 (45.37)	3,670 (55.95)	2,824 (43.05)	2,761 (42.06)	2,654 (40.44)	
IFG	11,442 (43.59)	2,547 (38.83)	2,956 (45.06)	2,895 (44.10)	3,044 (46.39)	
DM	2,896 (11.03)	343 (5.23)	780 (11.89)	909 (13.85)	864 (13.17)	

Based on the transitions in glycemic status, the population was categorized into three groups: reversion to normoglycemia (*n* = 11,909), persistent IFG (*n* = 11,442), and progression to DM (*n* = 2,896). As shown in Table 2, baseline characteristics demonstrated clear trends across the groups from reversion to normoglycemia, to persistent IFG, and to progression to DM. Specifically, there was a progressive increase in age, a higher proportion of males, increased BMI, and elevated baseline FPG levels. Furthermore, levels of TC, TG, and LDL-C progressively increased, while HDL-C levels decreased. Levels of SBP, DBP, and BUN, median levels of ALT and AST, and the proportion with a positive family history of DM also showed a worsening trend from the reversion group to the persistent IFG group and to the DM group (all *p* < 0.001). Significant differences were also observed in Scr levels, smoking, and drinking status among the groups (all *p* < 0.001). In contrast, no significant difference in height was found among the three groups (*p* = 0.384).

**Table 2:** Baseline demographic and clinical characteristics of the study cohort, stratified by glycemic status.

Variables	Reversion to normoglycemia (*n* = 11,909)	Persistence of IFG (*n* = 11,442)	Progression to DM (*n* = 2,896)	*p*-value
Age (years), Mean ± SD	45.50 ± 13.57	51.26 ± 13.34	55.50 ± 12.54	< 0.001
Gender, *n* (%)				< 0.001
Male	7,468 (62.71)	7,837 (68.49)	2,119 (73.17)	
Female	4,441 (37.29)	3,605 (31.51)	777 (26.83)	
BMI (kg/m^2^), mean ± SD	24.17 ± 3.32	25.13 ± 3.27	26.26 ± 3.37	< 0.001
Height (cm), mean ± SD	166.74 ± 8.40	166.62 ± 8.28	166.82 ± 8.34	0.384
Body weight (kg), mean ± SD	67.48 ± 12.13	70.02 ± 11.94	73.37 ± 12.54	< 0.001
CUN-BAE index, mean ± SD	27.25 ± 7.17	28.75 ± 6.94	30.31 ± 6.49	< 0.001
SBP (mmHg), mean ± SD	124.15 ± 16.71	129.11 ± 17.68	132.36 ± 18.48	< 0.001
DBP (mmHg), mean ± SD	76.76 ± 10.75	79.54 ± 11.19	80.83 ± 11.62	< 0.001
Baseline FPG (mmol/L), mean ± SD	5.83 ± 0.24	5.99 ± 0.32	6.29 ± 0.38	< 0.001
TC (mmol/L), mean ± SD	4.89 ± 0.95	5.03 ± 0.96	5.10 ± 0.96	< 0.001
TG (mmol/L), median (IQR)	1.28 (0.87, 1.91)	1.50 (1.02, 2.21)	1.77 (1.21, 2.60)	< 0.001
HDL-C (mmol/L), mean ± SD	1.34 ± 0.30	1.32 ± 0.29	1.29 ± 0.33	< 0.001
LDL-C (mmol/L), mean ± SD	2.84 ± 0.72	2.92 ± 0.72	2.93 ± 0.72	< 0.001
ALT (U/L), median (IQR)	20.20 (14.10, 31.00)	23.00 (16.00, 33.90)	26.90 (18.70, 42.00)	< 0.001
AST (U/L), median (IQR)	23.60 (19.50, 29.00)	24.00 (20.00, 30.00)	26.00 (21.10, 32.60)	< 0.001
BUN (mmol/L), mean ± SD	4.92 ± 1.24	5.04 ± 1.24	5.06 ± 1.27	< 0.001
Scr (μmol/L), mean ± SD	72.00 ± 16.22	73.51 ± 15.70	72.93 ± 16.09	< 0.001
Smoking status, *n* (%)				< 0.001
Current smoker	949 (7.97)	1,038 (9.07)	274 (9.46)	
Ever smoker	189 (1.59)	174 (1.52)	55 (1.90)	
Never smoker	3,060 (25.69)	2,634 (23.02)	472 (16.30)	
Not recorded	7,711 (64.75)	7,596 (66.39)	2095 (72.34)	
Drinking status, *n* (%)				< 0.001
Current drinker	135 (1.13)	179 (1.56)	45 (1.55)	
Ever drinker	743 (6.24)	656 (5.73)	142 (4.90)	
Never drinker	3,320 (27.88)	3,011 (26.32)	614 (21.2)	
Not recorded	7,711 (64.75)	7,596 (66.39)	2095 (72.34)	
Family history of DM, *n* (%)				< 0.001
No	11,650 (97.83)	11,175 (97.67)	2,780 (95.99)	
Yes	259 (2.17)	267 (2.33)	116 (4.01)	

**Figure 3:**
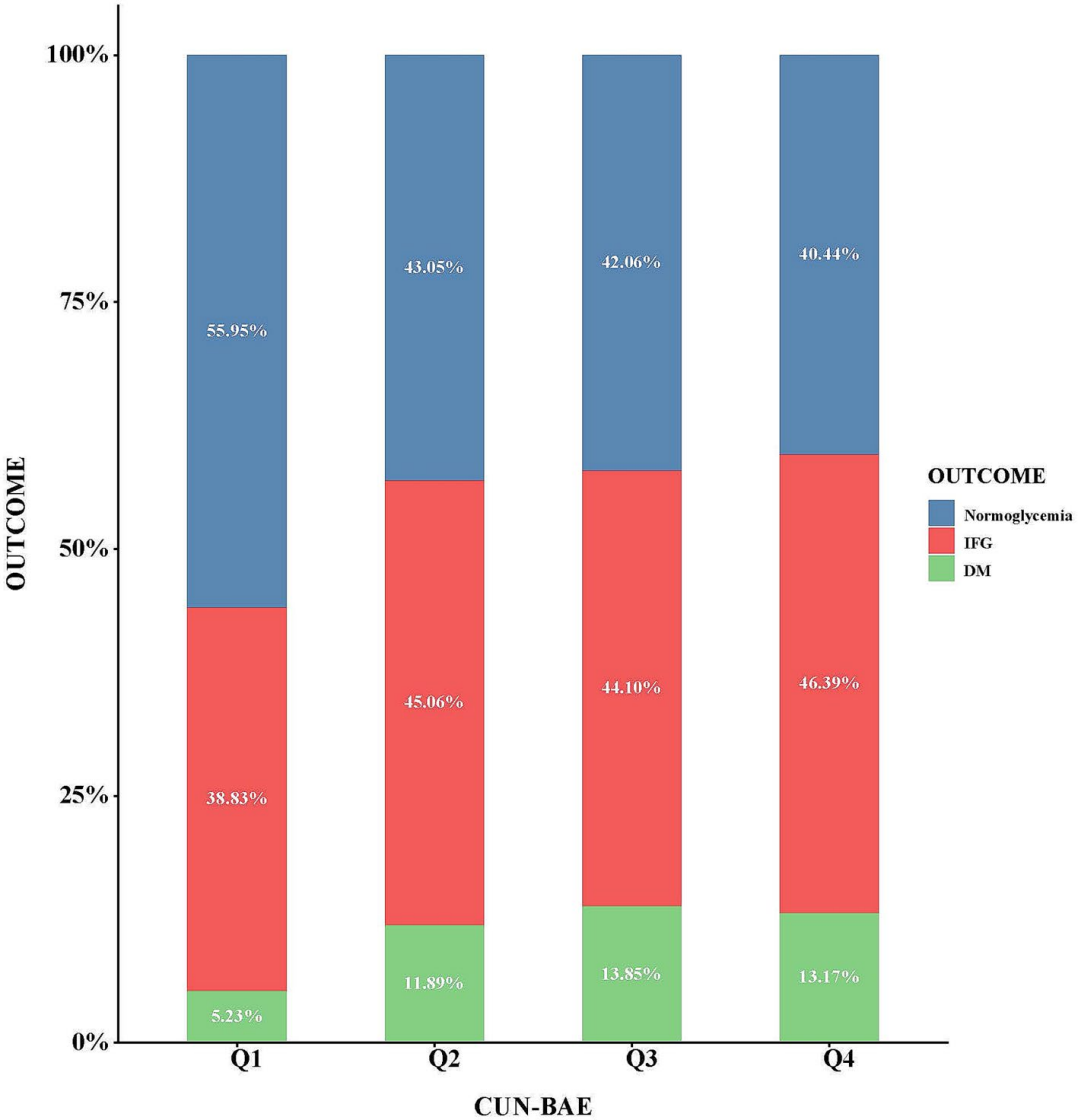
Distribution of glycemic outcomes stratified by baseline CUN-BAE index quartiles. Stacked bar charts showing the proportion of participants achieving normoglycemia (blue), IFG (red), and DM (green) across CUN-BAE quartiles (Q1–Q4). Proportions are presented as percentages.

Figure 3 illustrates the distribution of glycemic outcomes across baseline CUN-BAE index quartiles (Q1 – Q4). The stacked bars represent the proportion of participants achieving normoglycemia, IFG, and DM. A clear graded trend is observed: as the CUN-BAE quartile increases, the proportion of participants with normoglycemia decreases (from 55.95% in Q1 to 40.44% in Q4), while the proportions of both IFG and DM increase progressively. These findings indicate significant differences in glycemic status distribution among the four CUN-BAE index groups.

### Association between the CUN-BAE index and transitions in glycemic status

As shown in Table 3, the association between baseline CUN-BAE index and transitions in glycemic status was assessed using Cox proportional hazards regression models. For the outcome of reversion from IFG to normoglycemia, when analyzing the CUN-BAE index as a continuous variable, the HRs were all less than 1 in the crude model, Model I, and Model II (Crude model: HR 0.98, 95% CI 0.98–0.98; Model I: HR 0.99, 95% CI 0.99–0.99; Model II: HR 0.99, 95% CI 0.98–0.99; all *p* < 0.001). When the CUN-BAE index was analyzed by quartile groups (with Q1 as the reference), in Model II, the HRs for the Q2, Q3, and Q4 groups were 0.83 (95% CI 0.79–0.87), 0.82 (95% CI 0.78–0.87), and 0.82 (95% CI 0.77–0.87), respectively, with a trend test *p* < 0.001.

**Table 3:** Relationship of CUN-BAE index with glucose status transition in an IFG population assessed by cox proportional-hazards regression in multiple models.

Variables	Crude model		Model I		Model II	
	HR (95% CI)	*p*-value	HR (95% CI)	*p*-value	HR (95% CI)	*p*-value
IFG to normoglycemia						
CUN-BAE index	0.98 (0.98, 0.98)	< 0.001	0.99 (0.99, 0.99)	< 0.001	0.99 (0.98, 0.99)	< 0.001
(CUN-BAE index quartiles)
Q1	1.00 (Reference)		1.00 (Reference)		1.00 (Reference)	
Q2	0.70 (0.67, 0.73)	< 0.001	0.79 (0.75, 0.83)	< 0.001	0.83 (0.79, 0.87)	< 0.001
Q3	0.71 (0.68, 0.75)	< 0.001	0.81 (0.77, 0.85)	< 0.001	0.82 (0.78, 0.87)	< 0.001
Q4	0.72 (0.69, 0.76)	< 0.001	0.83 (0.79, 0.88)	< 0.001	0.82 (0.77, 0.87)	< 0.001
*P* for trend		< 0.001		< 0.001		< 0.001
IFG to DM						
CUN-BAE index	1.04 (1.04, 1.05)	< 0.001	1.03 (1.02, 1.04)	< 0.001	1.04 (1.03, 1.04)	< 0.001
(CUN-BAE index quartiles)
Q1	1.00 (Reference)		1.00 (Reference)		1.00 (Reference)	
Q2	1.98 (1.75, 2.25)	< 0.001	1.51 (1.33, 1.72)	< 0.001	1.47 (1.29, 1.68)	< 0.001
Q3	2.44 (2.15, 2.76)	< 0.001	1.79 (1.58, 2.03)	< 0.001	1.80 (1.59, 2.05)	< 0.001
Q4	2.52 (2.23, 2.86)	< 0.001	1.86 (1.63, 2.11)	< 0.001	2.02 (1.76, 2.31)	< 0.001
*P* for trend		< 0.001		< 0.001		< 0.001

Crude model: unadjusted. Model I: adjusted for FPG, SBP, DBP, and family history of DM at baseline. Model II: further adjusted for ALT, AST, LDL-C, HDL-C, TC, TG, BUN, Scr, smoking status, and drinking status at baseline.

For the outcome of progression from IFG to DM, when the CUN-BAE index was analyzed as a continuous variable, the HRs were greater than 1 in all models (Crude model: HR 1.04, 95% CI 1.04–1.05; Model I: HR 1.03, 95% CI 1.02–1.04; Model II: HR 1.04, 95% CI 1.03–1.04; all *p* < 0.001). Analysis by quartiles showed that in Model II, compared to the Q1 group, the Q2, Q3, and Q4 groups had a significantly increased risk of progression to DM, with HRs of 1.47 (95% CI 1.29–1.68), 1.8 (95% CI 1.59–2.05), and 2.02 (95% CI 1.76–2.31), respectively (trend test *p* < 0.001).

As shown in Table 4, the results of the logistic regression analysis were consistent with those of the Cox regression. For reversion from IFG to normoglycemia, when the CUN-BAE index was treated as a continuous variable, the OR in Model II was 0.97 (95% CI 0.96–0.97, *p* < 0.001). In the quartile-based analysis, the ORs for the Q2, Q3, and Q4 groups in Model II were 0.72 (95% CI 0.67–0.78), 0.69 (95% CI 0.64–0.74), and 0.58 (95% CI 0.53–0.63), respectively (trend test *p* < 0.001).

**Table 4:** Relationship of CUN-BAE index with glucose status transition in an IFG population assessed by logistic regression in multiple models.

Variables	Crude model		Model I		Model II	
	OR (95% CI)	*p*-value	OR (95% CI)	*p*-value	OR (95% CI)	*p*-value
IFG to normoglycemia						
CUN-BAE index	0.96 (0.96, 0.97)	< 0.001	0.97 (0.97, 0.98)	< 0.001	0.97 (0.96, 0.97)	< 0.001
(CUN-BAE index quartiles)
Q1	1.00 (Reference)		1.00 (Reference)		1.00 (Reference)	
Q2	0.60 (0.56, 0.64)	< 0.001	0.69 (0.64, 0.75)	< 0.001	0.72 (0.67, 0.78)	< 0.001
Q3	0.57 (0.53, 0.61)	< 0.001	0.69 (0.64, 0.74)	< 0.001	0.69 (0.64, 0.74)	< 0.001
Q4	0.53 (0.50, 0.57)	< 0.001	0.65 (0.60, 0.70)	< 0.001	0.58 (0.53, 0.63)	< 0.001
*P* for trend		< 0.001		< 0.001		< 0.001
IFG to DM						
CUN-BAE index	1.05 (1.04, 1.05)	< 0.001	1.03 (1.03, 1.04)	< 0.001	1.04 (1.03, 1.05)	< 0.001
(CUN-BAE index quartiles)
Q1	1.00 (Reference)		1.00 (Reference)		1.00 (Reference)	
Q2	2.45 (2.14, 2.79)	< 0.001	2.01 (1.75, 2.31)	< 0.001	1.86 (1.61, 2.14)	< 0.001
Q3	2.91 (2.56, 3.32)	< 0.001	2.29 (1.99, 2.63)	< 0.001	2.12 (1.84, 2.44)	< 0.001
Q4	2.75 (2.41, 3.13)	< 0.001	2.11 (1.83, 2.42)	< 0.001	2.15 (1.85, 2.50)	< 0.001
*P* for trend		< 0.001		< 0.001		< 0.001

Crude model: unadjusted. Model I: adjusted for FPG, SBP, DBP, and family history of DM at baseline. Model II: further adjusted for ALT, AST, LDL-C, HDL-C, TC, TG, BUN, Scr, smoking status, and drinking status at baseline.

For progression from IFG to DM, the OR for the CUN-BAE index as a continuous variable in Model II was 1.04 (95% CI 1.03–1.05, *p* < 0.001). The quartile analysis revealed that in Model II, the ORs for the Q2, Q3, and Q4 groups were 1.86 (95% CI 1.61–2.14), 2.12 (95% CI 1.84–2.44), and 2.15 (95% CI 1.85–2.50), respectively (trend test *p* < 0.001).

Sensitivity analyses in the Supplementary materials confirmed the robustness of the aforementioned associations. In both the original dataset containing all participants (Supplementary Tables 2, 3) and the dataset after excluding participants with incomplete covariate data (Supplementary Tables 4, 5), the direction and significance of the associations between the CUN-BAE index and the two glycemic transition outcomes were consistent with the main analysis results for both Cox and logistic regression models. Additionally, a stringent sensitivity analysis (Model III, adjusted for SBP, DBP, FPG, TC, TG, ALT, BUN, Scr, and family history of DM at baseline) adjusting exclusively for baseline covariates with complete or near-complete data yielded virtually identical results, confirming that the imputation of variables with higher missing rates did not artificially drive the observed associations (Supplementary Table 6). Furthermore, when the baseline IFG population was redefined according to the WHO criteria (FPG 6.1–6.9 mmol/L, *n* = 7,193), parallel sensitivity analyses (Supplementary Tables 7, 8) consistently demonstrated that higher CUN-BAE index levels remained significantly associated with adverse glycemic transitions, affirming the robustness of our findings across different definitional frameworks. Finally, to rigorously account for potential competing risk biases, the Fine-Gray subdistribution hazard models were applied. The derived subdistribution hazard ratios (SHRs) (Supplementary Table 9) were highly consistent in both direction and magnitude with the cause-specific HRs obtained from the standard Cox models, confirming that the presence of competing events did not substantially bias the observed associations.

### K-M survival analysis

**Figure 4:**
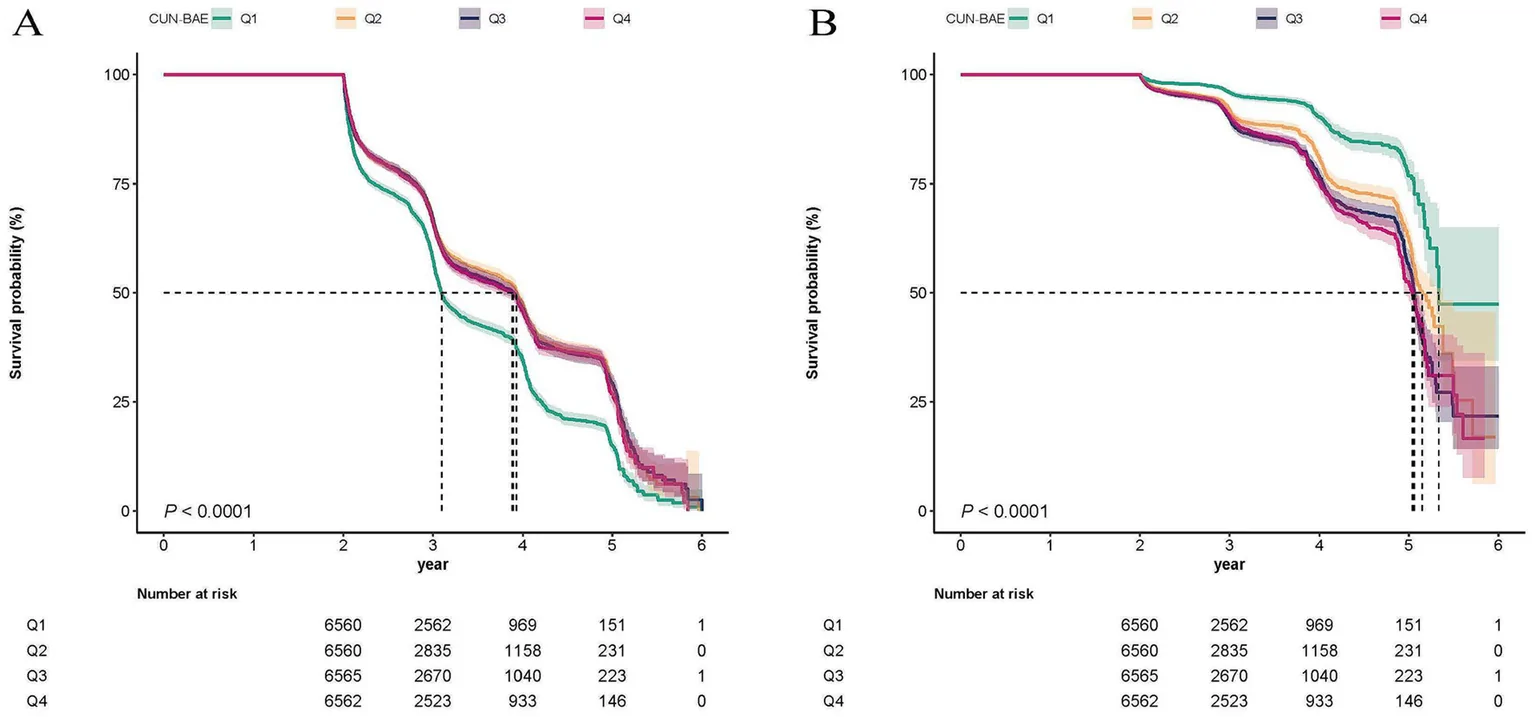
K-M curves for reversion to normoglycemia and progression to DM, stratified by CUN-BAE index quartiles, in an IFG population. **(A)** Reversion to normoglycemia. **(B)** Progression to diabetes.

Figure 4 presents K-M curves stratified by CUN-BAE index quartiles to assess the association between baseline CUN-BAE index levels and the risk of glycemic status transitions in individuals with IFG.

For the outcome of reversion to normoglycemia, the K-M curves demonstrated differences in the probability of reversion across CUN-BAE index quartile groups. The Q1 had the highest probability of reversion, while the Q4 had the lowest, indicating that a higher baseline CUN-BAE index was associated with a lower probability of reversion. The log-rank test showed a statistically significant difference in the cumulative incidence of normoglycemic reversion among the CUN-BAE index quartile groups (*p* < 0.001).

For the outcome of progression to DM, the K-M curves exhibited an opposite trend. The Q4 had the highest cumulative incidence of DM, while the Q1 had the lowest, suggesting that a higher baseline CUN-BAE index was associated with an increased risk of DM progression. The log-rank test revealed statistically significant differences in the cumulative incidence curves of DM among the CUN-BAE index quartile groups (*p* < 0.001).

### Analysis of nonlinear relationships and threshold effects between CUN-BAE index and glycemic status transitions

**Figure 5:**
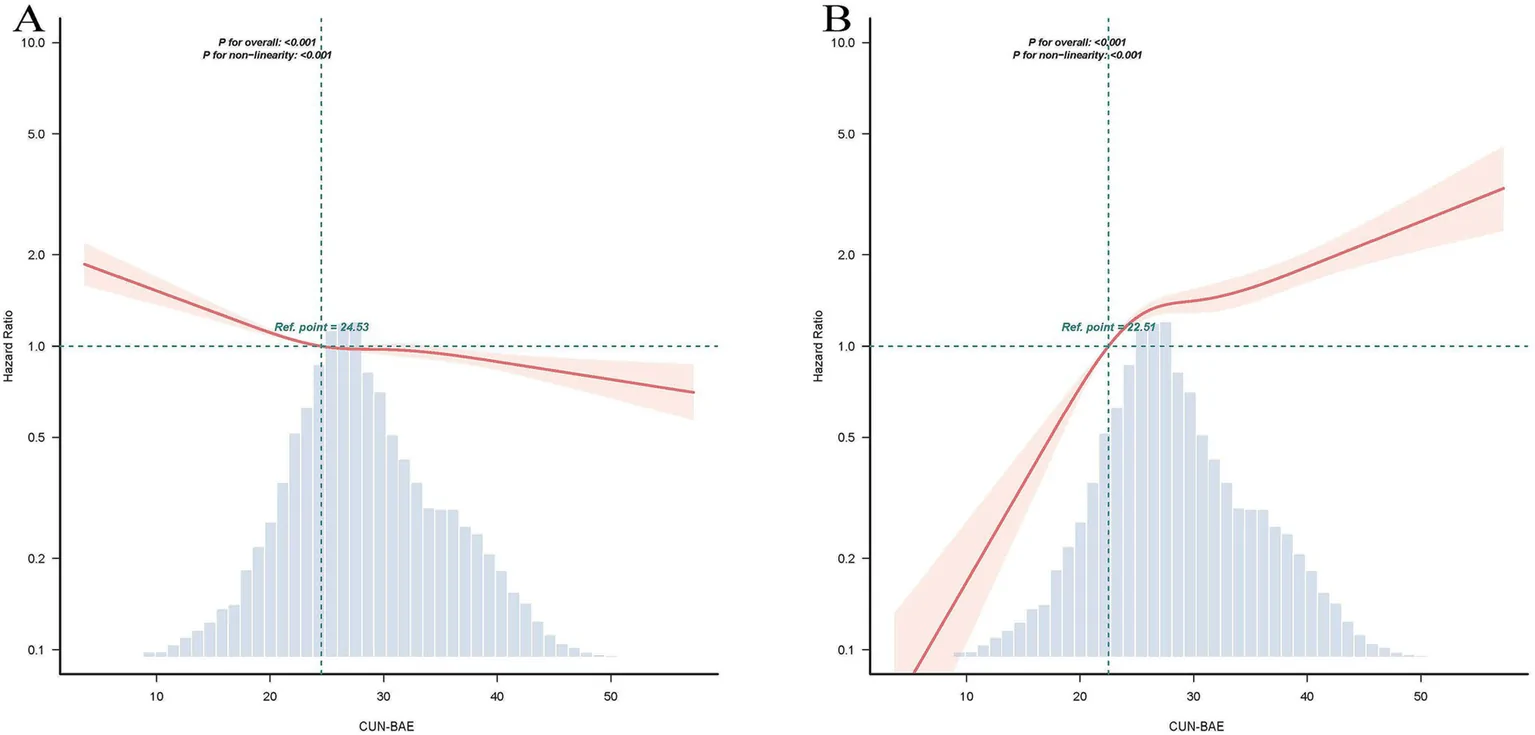
The non-linear relationship between the CUN-BAE index and incident normoglycemic/DM conversion in an IFG population. The relationship between the CUN-BAE index and incident normoglycemic/DM conversion in an IFG population. A non-linear association between CUN-BAE index and incident normoglycemic **(A)**, DM conversion **(B)** was found. Adjusted for FPG, SBP, DBP, ALT, AST, LDL-C, HDL-C, TC, TG, BUN, Scr, smoking status, and family history of DM at baseline.

The nonlinear relationships between CUN-BAE index and glycemic status transitions were analyzed using RCS models. Figure 5 displays the association curves between CUN-BAE index and the probability of reversion to normoglycemia (Panel A) and progression to DM (Panel B) in individuals with IFG. The curve-fitting results revealed a nonlinear association between CUN-BAE index and both glycemic transition outcomes (*P* for nonlinearity < 0.001). Specifically, the CUN-BAE index showed a negative correlation with the probability of reversion to normoglycemia and a positive correlation with the risk of progression to DM, with these associations not following a simple linear pattern.

To further evaluate the nonlinear relationships, a threshold effect analysis was performed. The results (Table 5) indicated statistically significant threshold effects of the CUN-BAE index on both glycemic transition outcomes. For the outcome of reversion from IFG to normoglycemia, an inflection point was identified at a CUN-BAE index value of 24.53. On either side of this threshold, the strength of the association between CUN-BAE index and the risk of reversion differed: when CUN-BAE index was below 24.53, the HR was 0.97 (95% CI, 0.96–0.98, *p* < 0.001); when CUN-BAE index was equal to or above 24.53, the HR was 0.99 (95% CI, 0.99–1.0, *p* = 0.0149). The likelihood ratio test for this threshold effect was significant (*p* < 0.001).

**Table 5:** Threshold effect analysis of CUN-BAE index on glucose status conversion in an IFG population.

CUN-BAE index	HR (95% CI)	*p*-value
IFG to normoglycemia		
< 24.53	0.97 (0.96, 0.98)	< 0.001
≥ 24.53	0.99 (0.99, 1.0)	0.0149
P for log-likelihood ratio test		< 0.001
IFG to DM		
< 22.51	1.14 (1.07, 1.22)	< 0.001
≥ 22.51	1.029 (1.02, 1.04)	< 0.001
*P* for log-likelihood ratio test		< 0.001

Adjusted for FPG, SBP, DBP, ALT, AST, LDL-C, HDL-C, TC, TG, BUN, Scr, smoking status, and family history of DM at baseline.

For the outcome of progression from IFG to DM, the analysis identified an inflection point at a CUN-BAE index value of 22.51. When the CUN-BAE index was below 22.51, the HR was 1.14 (95% CI: 1.07–1.22, *p* < 0.001); when the CUN-BAE index was equal to or above 22.51, the HR was 1.029 (95% CI, 1.02–1.04, *p* < 0.001). The likelihood ratio test was also significant (*p* < 0.001), confirming the existence of this threshold effect.

### Subgroup analysis

**Figure 6:**
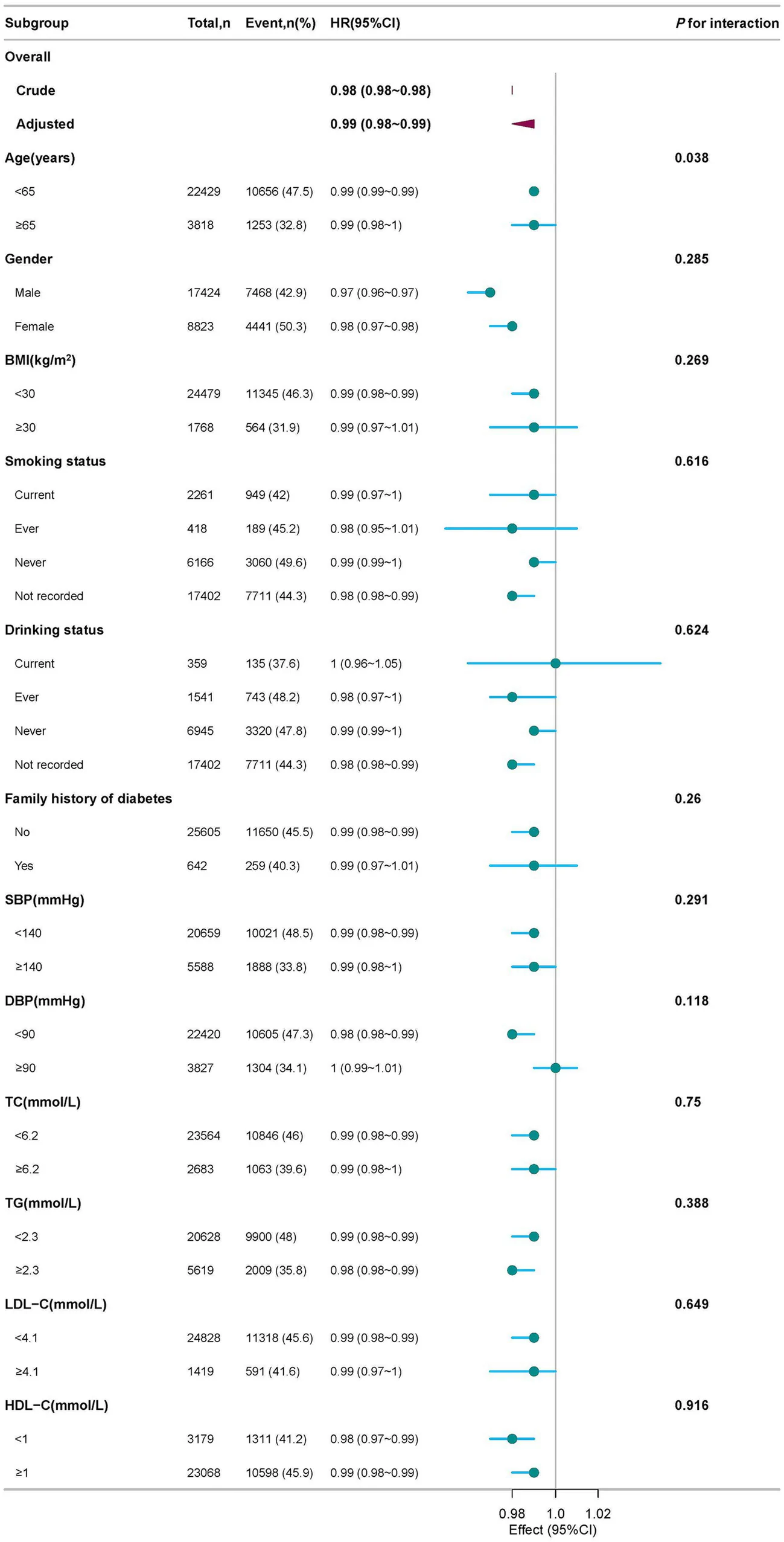
Forest plot of subgroup analysis for the association between CUN-BAE index and reversion to normoglycemia. Stratification was performed according to age, gender, BMI, SBP, DBP, TG, TC, LDL-C, HDL-C, smoking status, drinking status, and family history of DM. Models were adjusted for FPG, SBP, DBP, ALT, AST, LDL-C, HDL-C, TC, TG, BUN, Scr, smoking status, and family history of DM at baseline, excluding the stratification variable within each respective subgroup.

To assess the consistency of the association between CUN-BAE index and transitions in glycemic status across different population characteristics, we conducted subgroup analyses, with the results presented as forest plots. Figure 6 displays the results of the subgroup analysis for the association between CUN-BAE index and reversion from IFG to normoglycemia. The analysis was stratified by age, gender, BMI, SBP, DBP, TG, TC, LDL-C, HDL-C, smoking status, drinking status, and family history of DM. All models were further adjusted for baseline FPG, SBP, DBP, ALT, AST, LDL-C, HDL-C, TC, TG, BUN, Scr, smoking status, drinking status, and family history of DM, with the stratification variable itself excluded from adjustment within its respective stratum. The forest plot results indicate that the inverse association between the CUN-BAE index and reversion to normoglycemia was generally consistent across all subgroups.

**Figure 7:**
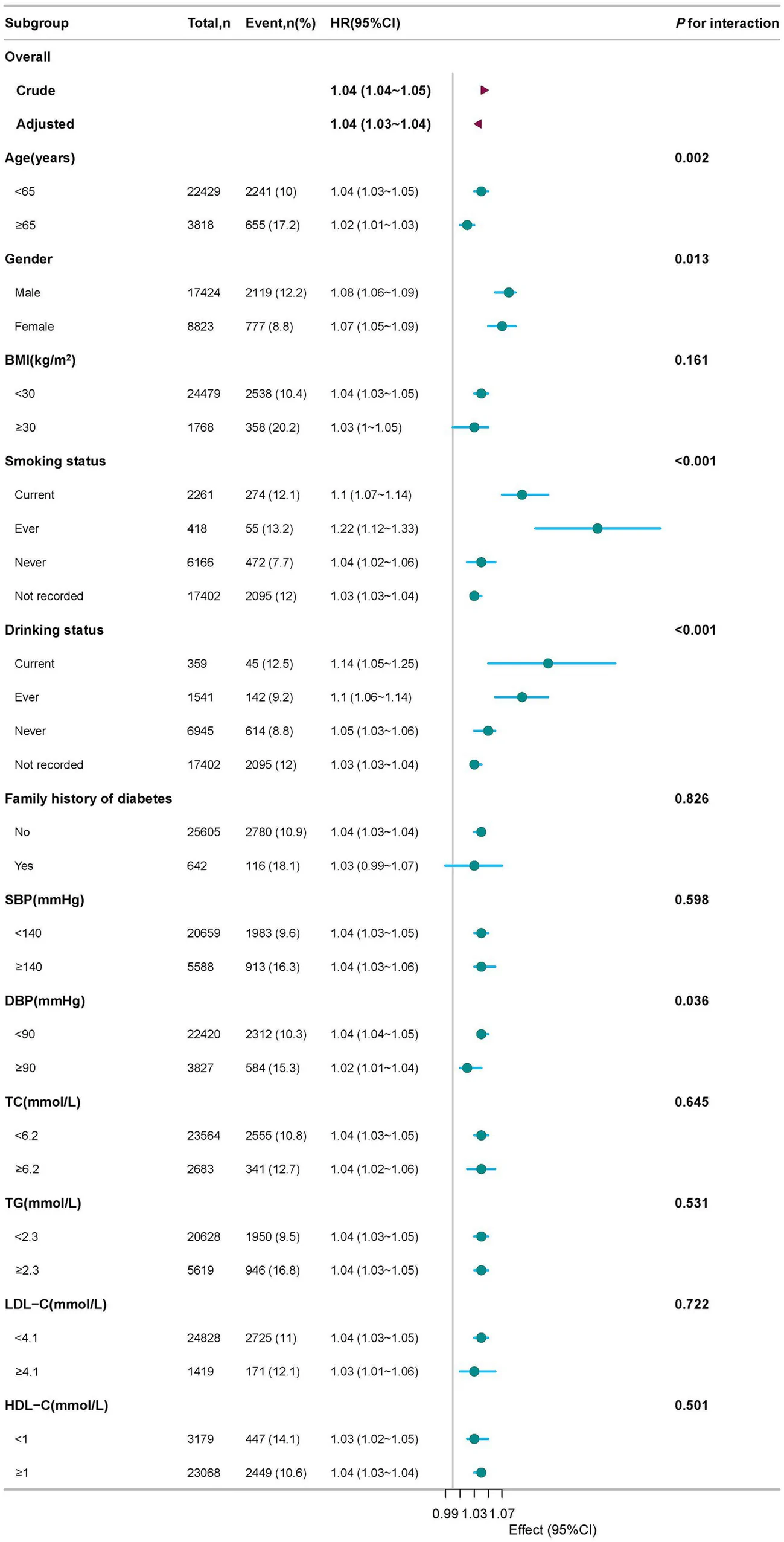
Forest plot of subgroup analysis for the association between CUN-BAE index and progression to DM. Stratification was performed according to age, gender, BMI, SBP, DBP, TG, TC, LDL-C, HDL-C, smoking status, drinking status, and family history of DM. Models were further adjusted for baseline FPG, SBP, DBP, ALT, AST, LDL-C, HDL-C, TC, TG, BUN, Scr, smoking status, and family history of DM, excluding the stratification variable within each respective subgroup.

Figure 7 presents the results of the subgroup analysis for the association between CUN-BAE index and progression from IFG to DM. The stratification variables and adjustment factors were the same as in Figure 6. The forest plot results show that the positive association between CUN-BAE index and progression to DM was largely consistent across all subgroups.

## Discussion

The primary objective of this study was to investigate whether the CUN-BAE index was associated with future transitions in glycemic status among individuals with IFG within a large, multicenter Chinese cohort. This retrospective analysis of 26,247 individuals yielded several main findings. First, a significant and consistent dose–response relationship was observed between baseline CUN-BAE index levels and glycemic outcomes. Specifically, higher CUN-BAE index values were independently associated with a lower probability of reversion to normoglycemia and a higher risk of incident DM. Second, our study revealed significant nonlinear associations and threshold effects between the CUN-BAE index and both bidirectional glycemic trajectories. These findings suggest the existence of critical tipping points regarding the impact of body fat accumulation on glucose metabolism. Finally, the robustness and consistency of these associations were confirmed through comprehensive sensitivity analyses—including validation across different IFG definitional frameworks (ADA *vs.* WHO)—and subgroup analyses encompassing key demographic and clinical characteristics. Because IFG represents a crucial crossroad where individuals may either revert to normoglycemia or progress to DM, identifying key associated factors that influence this bidirectional transition is essential for implementing precise prevention strategies. Ultimately, the CUN-BAE index, serving as a reliable surrogate for body fat content, provides valuable insights into the risk of glycemic transitions in the IFG population.

The results of this study clearly demonstrate that a higher baseline CUN-BAE index—an estimator of body fat percentage (BF%)—is an independent and robust risk marker for unfavorable glycemic transitions in individuals with IFG. Its association with glycemic outcomes exhibits a clinically meaningful gradient: higher CUN-BAE index levels are associated with a lower likelihood of reversion to normoglycemia and a higher risk of progression to DM. This finding reinforces the central role of excess adiposity in the natural history of prediabetes (25). More importantly, our study advances this association from the traditional metric of BMI to the more specific level of body fat estimation. Compared to BMI, the CUN-BAE index is optimized by incorporating the influence of age and gender on BF%, providing a more accurate reflection of an individual’s true adiposity burden (26). Therefore, the findings suggest that within the IFG population, excessive accumulation of body fat proportion may be a more critical factor driving the deterioration of glucose metabolism than general overweight/obesity reflected by BMI. This explains why the association between obesity and DM risk might have been underestimated or shown heterogeneity in some prior studies relying solely on BMI (27). A key finding of this study is the significant nonlinear relationship and threshold effect between the CUN-BAE index and the two glycemic transition outcomes. For DM progression, the risk gradient was particularly steep (HR 1.14) when the CUN-BAE index was below 22.51. Beyond this threshold, the risk continued to increase, but the trend became more attenuated (HR 1.029). This nonlinear pattern may suggest that in the early stages of fat accumulation, adipose tissue function remains relatively adaptable, and the degree of metabolic disturbance is approximately linearly related to the increase in fat mass. However, once adiposity exceeds a certain critical point (the threshold), it may trigger significant adipose tissue dysfunction, such as adipocyte hypertrophy reaching its limit, exacerbated hypoxia, and macrophage infiltration leading to chronic inflammation (28–30). Beyond this point, even with further increases in adiposity, the marginal detrimental effect on metabolism might be relatively weaker, although the overall risk remains elevated. Similarly, the threshold for reversion to normoglycemia carries important implications: below this level, the glycemic benefit from reducing adiposity might be particularly pronounced. This offers a potential, quantifiable target for body fat management in clinical practice, suggesting that reducing the CUN-BAE index of IFG patients below this threshold may significantly increase the chance of glycemic reversion. From a pathophysiological perspective, the findings align with the central mechanism of insulin resistance induced by dysfunctional adipose tissue. Excess body fat, particularly visceral fat, impairs insulin signaling in the liver and muscles through pathways such as increased free fatty acid release and the secretion of various adipokines and pro-inflammatory cytokines (e.g., TNF-*α* and IL-6) (31–33). The elevated total adiposity reflected by CUN-BAE index likely represents a composite manifestation of this cascade of detrimental metabolic processes. The data in Table 1 of this study, showing a parallel worsening of baseline FPG, BP, TG, and other metabolic parameters with increasing CUN-BAE index quartiles, indirectly supports a close link between CUN-BAE index and insulin resistance and its associated metabolic abnormalities. Consequently, a higher CUN-BAE index likely identifies a state of more severe insulin resistance, making *β*-cell compensatory function more prone to exhaustion. This in turn increases the risk of DM development and reduces the likelihood of spontaneous reversion to normoglycemia.

Our finding that a higher baseline CUN-BAE index is independently associated with a reduced probability of reversion to normoglycemia and an increased risk of progression to DM in individuals with IFG is consistent with and significantly extends the growing body of evidence. As synthesized in the Background, while previous large-scale studies by Peng et al. (20), Dominguez et al. (22), and Geng et al. (21) established the robust link between higher CUN-BAE levels and incident DM in general populations, our study provides crucial novel evidence specifically tailored to the high-risk IFG cohort. A key innovation of our analysis lies in exploring bidirectional glycemic outcomes within the same population. By demonstrating that the degree of adiposity estimated by CUN-BAE is equally strongly related to the likelihood of achieving normoglycemia, our dual perspective offers a more comprehensive understanding of the metabolic trajectory in prediabetes. Furthermore, the significant nonlinear relationships and threshold effects we identified for risk stratification represent a key distinction from earlier work (18). This nonlinear pattern suggests that beyond a critical point, the detrimental metabolic impact of excess adiposity may accelerate, a concept supported by pathophysiological models linking severe obesity to accelerated insulin resistance and *β*-cell dysfunction (32). The superior performance of CUN-BAE index over BMI aligns with its fundamental design principle: to provide a more accurate estimate of actual body fat by incorporating the influences of age and gender. This is particularly important in the IFG population, where the metabolic consequences of body fat may be more critical than overall body size. While recent studies have validated CUN-BAE as an effective risk marker for other cardiometabolic risks, such as metabolic dysfunction-associated steatotic liver disease (MASLD) (34) and hypertension-related diabetes (26), our findings provide quantifiable adiposity targets for personalized intervention strategies specifically in clinical IFG management. In summary, by elucidating its specific association with bidirectional glycemic outcomes in a well-defined IFG cohort and uncovering critical threshold effects, our study addresses an important evidence gap and highlights the potential of the CUN-BAE index to enhance risk stratification and early intervention for this large and vulnerable population.

The findings of this study carry significant clinical implications. Firstly, this research confirms the bidirectional association of the CUN-BAE index—a simple tool estimating BF% based on BMI, age, and gender—for glycemic transitions in individuals with IFG, a critical prediabetic stage. This not only expands the application scenarios for the CUN-BAE index but, more importantly, the revealed significant nonlinear relationships and precise threshold effects provide unprecedented quantitative evidence for the precise management of IFG. Specifically, the threshold points identified in this study represent the core clinical value. For glycemic reversion, the inverse association with the probability of reversion was stronger (HR 0.97) when the CUN-BAE index was below 24.53. This suggests that for IFG individuals with adiposity levels below this threshold, lifestyle interventions aimed at reducing body fat hold greater potential benefit for achieving normoglycemia. Clinicians should encourage and reinforce weight management in this subgroup. Conversely, when the CUN-BAE index exceeds 24.53, the association weakens (HR 0.99), indicating increased difficulty in achieving reversion and potentially necessitating more intensive, comprehensive management strategies. For DM progression, the identified threshold (CUN-BAE index = 22.51) holds even greater early-warning value. Below this threshold, each unit increase in the CUN-BAE index was associated with a sharp 14% increase in DM risk (HR 1.14), marking a phase of accelerated risk. This finding emphasizes that clinical attention should not be limited to IFG individuals meeting traditional obesity criteria. Those with adiposity levels just above this critical point, who may be classified as having “normal weight” or “overweight” by BMI, already have a significantly elevated risk of progression and should be considered a very high-risk population requiring early and active intervention. Beyond this threshold, the risk continued to increase with higher adiposity, but the trend plateaued (HR 1.029), suggesting their metabolic environment may have entered a relatively stable, high-risk plateau phase. Regarding practical application, the value of this study lies in advancing risk stratification from a qualitative assessment of “whether obese” to a quantitative, individualized stratification of “what level of adiposity corresponds to what level of risk.” The CUN-BAE index is calculated simply using routine examination data (height, weight, age, and gender), making it highly suitable for use as a primary risk stratification tool for IFG individuals in primary care settings and large-scale population screening. Based on the thresholds established here, a concise clinical decision pathway can be constructed. For example, IFG individuals with CUN-BAE index < 22.51 could be assigned to routine management; those with CUN-BAE index between 22.51 and 24.53 could be prioritized for intensive intervention, aiming to reduce their adiposity below 22.51 to minimize progression risk; and individuals with CUN-BAE index > 24.53 may require comprehensive management plans, including consideration of pharmacological interventions, to pursue glycemic reversion. Furthermore, the results of this study are highly congruent with recent literature, collectively establishing the superiority of CUN-BAE index in metabolic risk assessment. Multiple studies have indicated that the CUN-BAE index outperforms traditional BMI in assessing the risk of DM, metabolic syndrome, and cardiovascular disease (20, 21, 23). It holds particular, irreplaceable value in identifying individuals with metabolically obese normal weight (MONW) —those with normal BMI but excess body fat. Building upon this foundation, our study provides specific CUN-BAE index cut-off points for risk stratification of glycemic transitions in the IFG population, enabling more precise clinical application of this metric. In conclusion, this study establishes the CUN-BAE index as a strong associated factor of glycemic transitions in individuals with IFG. The identified threshold effects provide crucial scientific evidence for formulating individualized adiposity management targets and intervention intensities. Future research directions include validating the guiding value of these thresholds in prospective intervention trials and combining the CUN-BAE index with emerging metabolic markers to build more comprehensive risk assessment models, ultimately providing more precise tools for the primary prevention of DM.

This study possesses several methodological strengths that significantly enhance the reliability and generalizability of the findings. First, the analysis was based on a large-scale, multicenter Chinese cohort, incorporating a total of 26,247 individuals with IFG at baseline, followed by retrospective analysis and long-term follow-up. The large sample size ensured sufficient statistical power and reduced the risk of random error, while the multicenter design helped mitigate potential selection bias inherent to single-center studies, making the results more representative of the broader IFG population. Second, the study employed rigorous and comprehensive statistical methods. We not only utilized multivariable Cox proportional hazards regression models to assess the association between CUN-BAE index and glycemic transitions but also performed multiple imputation to handle missing data, ensuring dataset completeness and reducing associated bias. Furthermore, extensive sensitivity analyses confirmed the robustness of the primary results. Importantly, the application of RCS allowed for the identification of nonlinear relationships and precise threshold effects between the CUN-BAE index and the glycemic outcomes, providing crucial early-warning information for risk stratification. Finally, the robustness and generalizability of the findings were further strengthened through extensive subgroup analyses and interaction tests. These subgroup analyses covered key demographic and clinical characteristics, including different age groups, genders, baseline BMI, BP, and lipid levels. The results consistently demonstrated the same direction of association between the CUN-BAE index and glycemic transitions across these subgroups. This indicates that the association of the CUN-BAE index with glycemic outcomes is broadly applicable across different subpopulations of individuals with IFG.

While this study, through its large-scale multicenter design and rigorous statistical methods, provides robust evidence regarding the association between the CUN-BAE index and glycemic outcomes, several limitations must be acknowledged. First, the retrospective study design is inherently susceptible to potential residual confounding. Although we controlled for known confounders through multivariable adjustment and sensitivity analyses, unmeasured variables—such as dietary patterns, levels of physical activity, and medication adherence—could influence glycemic transitions and thus introduce potential bias. This limitation is common to many observational studies and has been widely discussed in similar retrospective cohort studies utilizing large databases. Second, as a secondary data analysis, variable definitions and measurements were dependent on the specifications of the original dataset, which was not specifically designed for validating the CUN-BAE index. The diagnosis of IFG and diabetes in our study relied solely on FPG levels and self-reported medical history, without the availability of oral glucose tolerance tests or glycated hemoglobin (HbA1c) measurements. Consequently, a proportion of individuals with isolated postprandial hyperglycemia might have been misclassified as having IFG at baseline, which could introduce potential selection bias. Furthermore, while weight, height, and glycemic indices were measured using standardized protocols, more detailed body composition data—such as body fat distribution and muscle mass—were not available. This may limit a deeper mechanistic exploration of the pathways underlying the observed associations. Although leveraging existing health record data is efficient, it is inevitably constrained by the scope and precision of the original data collection. Third, the specificity of the study population may affect the generalizability of the results. The cohort consisted exclusively of Chinese adults with baseline IFG. It is important to note that the CUN-BAE index was originally developed in a Caucasian population. Given the well-established influence of racial and regional factors on body fat distribution, caution is warranted when interpreting the absolute values of the estimated body fat. While this design enhanced internal homogeneity, extrapolating the findings to other ethnicities or normoglycemic populations requires further investigation. However, it should be emphasized that the present study is primarily an association analysis designed to explore the relative relationship between CUN-BAE index levels and glycemic transitions rather than to validate its absolute diagnostic accuracy. Furthermore, recent studies have increasingly supported the reliable application of the CUN-BAE index in Asian populations, demonstrating its robust utility in evaluating risks for prediabetes, metabolic disorders, and adverse clinical outcomes. Future validation in more diverse populations with direct body composition measurements is necessary to confirm the broad applicability of the CUN-BAE index. Finally, although multiple imputation under the MAR assumption was used to handle missing data, it is important to acknowledge that several variables had missing rates exceeding 15%. To mitigate this, we performed a rigorous sensitivity analysis (Model III) excluding these highly missing variables, which demonstrated that the fundamental directional associations remained robust, thereby minimizing the likelihood that imputation artifacts drove our primary conclusions. Moreover, although the complete-case cohort yielded consistent primary outcomes, this subset may represent a systematically different subpopulation—likely individuals who opted for more comprehensive health evaluations—compared to the overall imputed sample. Consequently, the potential for residual bias due to discrepancies between the complete-case and overall cohorts, or unobserved non-random missingness, cannot be entirely ruled out. Furthermore, while the CUN-BAE index offers the advantage of being a simple, calculated index, it remains an estimator of BF% rather than a direct measurement. Although it correlates well with gold-standard methods, estimation error may affect precision in risk assessment at the individual level. Finally, although multiple imputation was used to handle missing data, and loss to follow-up was verified to have minimal impact on the primary outcomes, the potential for bias due to non-random attrition cannot be entirely ruled out.

Building upon the findings and limitations of this study, future research should advance in four key directions to validate and translate these conclusions. First, large-scale, multicenter prospective cohort studies are warranted to overcome the constraints of the present retrospective design. Systematically collecting data on unmeasured confounders (such as dietary patterns and physical activity) and incorporating repeated CUN-BAE index measurements will enable a more robust assessment of the longitudinal association between adiposity trajectories and IFG progression. Second, mechanistic sub-studies should be conducted using precise body composition assessments, such as dual-energy X-ray absorptiometry (DXA) or bioelectrical impedance analysis (BIA), alongside measurements of metabolic and inflammatory biomarkers. This approach will help elucidate the physiological pathways linking the CUN-BAE index to altered glucose metabolism. Third, external validation across populations with diverse demographic backgrounds and in various clinical settings is essential to confirm the global generalizability of the identified CUN-BAE index thresholds. Finally, the ultimate goal is to design randomized controlled trials to evaluate the clinical effectiveness of CUN-BAE-guided interventions. By comparing glycemic outcomes between patients receiving risk-stratified management based on these thresholds and those receiving usual care, future studies can definitively translate this index from an epidemiological marker into a practical decision-making tool for precision prediabetes management.

## Conclusion

This study, within a large-scale, multicenter cohort of Chinese adults with IFG, confirms that the baseline CUN-BAE index is independently associated with transitions in glycemic status. A higher CUN-BAE index value was significantly associated with a lower probability of reversion to normoglycemia and a concurrently higher risk of incident DM. This association exhibited a nonlinear pattern with clear threshold effects, highlighting the pivotal role of body fat management in determining glycemic outcomes among individuals with IFG. The findings reinforce the potential clinical utility of the CUN-BAE index as a convenient and effective tool for estimating adiposity in identifying high-risk individuals within the prediabetic population. Compared to the traditional BMI, the CUN-BAE index provides a more precise reflection of an individual’s true adiposity burden, thereby offering a critical foundation for early, precise risk stratification and personalized intervention strategies. Future research should focus on prospectively validating the clinical utility of the CUN-BAE index thresholds identified in this study and exploring the integration of this metric into existing DM prevention guidelines and clinical decision pathways, with the ultimate aim of improving long-term health outcomes for the IFG population.

## Data Availability

Publicly available datasets were analyzed in this study. This data can be found here: the dataset presented in this study is available in the online repository (https://doi.org/10.5061/dryad.ft8750v).

## Footnotes

1
https://doi.org/10.5061/dryad.ft8750v

## Glossary

ANOVA: One-way analysis of variance
ADA: American Diabetes Association
ALT: Alanine aminotransferase
AST: Aspartate aminotransferase
BF%: Body fat percentage
BMI: Body mass index
BP: Blood pressure
BUN: Blood urea nitrogen
CI: Confidence interval
CUN-BAE: Clínica Universidad de Navarra-Body Adiposity Estimator
DBP: Diastolic blood pressure
DM: Diabetes Mellitus
FPG: Fasting plasma glucose
HDL-C: High-density lipoprotein cholesterol
HR: Hazard ratio
IFG: Impaired fasting glucose
IQR: Interquartile range
K-M: Kaplan–Meier
LDL-C: Low-density lipoprotein cholesterol
MASLD: Metabolic dysfunction-associated steatotic liver disease
MICE: Multiple imputation by Chained equations
MONW: Metabolically obese normal weight
OR: Odds ratio
RCS: Restricted cubic spline
SBP: Systolic blood pressure
Scr: Serum creatinine
SD: Standard deviation
TC: Total cholesterol
TG: Triglycerides
TNF-*α*: Tumor necrosis factor-α
IL-6: Interleukin-6
DXA: Dual-energy X-ray absorptiometry
BIA: Bioelectrical impedance analysis
STROBE: Strengthening the reporting of observational studies in epidemiology
HbA1c: Glycated hemoglobin
WHO: World Health Organization
