# Supplementary material for: Association of the CUN-BAE index with glycemic outcomes in individuals with impaired fasting glucose: a retrospective multicenter Chinese cohort study
Source: Front Nutr. 2026 Sep 10;13:1813094. doi: 10.3389/fnut.2026.1813094 (PMC13600847; doi:10.3389/fnut.2026.1813094)
Supplement: Supplementary file 1 [file Table_1.docx]

**Supplementary Table 1. Description of missing data.**

| **Variables** | **Non-missing** | **Missing** | **Miss percentage(%)** |
| --- | --- | --- | --- |
| Age | 26247 | 0 | 0 |
| Gender | 26247 | 0 | 0 |
| Height | 26247 | 0 | 0 |
| Body weight | 26247 | 0 | 0 |
| BMI | 26247 | 0 | 0 |
| Baseline FPG | 26247 | 0 | 0 |
| ALT | 26010 | 237 | 0.903 |
| AST | 11397 | 14850 | 56.578 |
| BUN | 23376 | 2871 | 10.938 |
| Scr | 24896 | 1351 | 5.147 |
| SBP | 26240 | 7 | 0.027 |
| DBP | 26240 | 7 | 0.027 |
| HDL-C | 15574 | 10673 | 40.664 |
| LDL-C | 16208 | 10039 | 38.248 |
| TC | 25631 | 616 | 2.347 |
| TG | 25629 | 618 | 2.355 |
| Family histroy of DM | 26247 | 0 | 0 |
| Drinking status | 8845 | 17402 | 66.301 |
| Smoking status | 8845 | 17402 | 66.301 |

**Supplementary Table 2. Relationship of CUN-BAE index with** **glycemic status conversion in an IFG population assessed by Cox proportional-hazards regression in multiple models (the original dataset).**

| Variables | Crude model | | Model Ⅰ | | Model Ⅱ | |
| --- | --- | --- | --- | --- | --- | --- |
|  | HR (95%CI) | *P*-Value | HR (95%CI) | *P*-Value | HR (95%CI) | *P*-Value |
| IFG to normoglycemia | | |  |  |  |  |
| CUN-BAE index | 0.98 (0.98, 0.98) | < 0.001 | 0.99 (0.99, 0.99) | < 0.001 | 0.97 (0.97, 0.98) | < 0.001 |
| (CUN-BAE index quartiles) |  |  |  |  |  |  |
| Q1 | 1.00 (Reference) |  | 1.00 (Reference) |  | 1.00 (Reference) |  |
| Q2 | 0.70 (0.67, 0.73) | < 0.001 | 0.79 (0.75, 0.83) | < 0.001 | 0.70 (0.63, 0.77) | < 0.001 |
| Q3 | 0.71 (0.68, 0.75) | < 0.001 | 0.81 (0.77, 0.85) | < 0.001 | 0.71 (0.64, 0.79) | < 0.001 |
| Q4 | 0.72 (0.69, 0.76) | < 0.001 | 0.83 (0.79, 0.88) | < 0.001 | 0.67 (0.60, 0.76) | < 0.001 |
| *P* for trend |  | < 0.001 |  | < 0.001 |  | < 0.001 |
| IFG to DM | | |  |  |  |  |
| CUN-BAE | 1.04 (1.04, 1.05) | < 0.001 | 1.03 (1.02, 1.04) | < 0.001 | 1.05 (1.04, 1.06) | < 0.001 |
| (CUN-BAE index quartiles) |  |  |  |  |  |  |
| Q1 | 1.00 (Reference) |  | 1.00 (Reference) |  | 1.00 (Reference) |  |
| Q2 | 1.98 (1.75, 2.25) | < 0.001 | 1.51 (1.33, 1.72) | < 0.001 | 1.36 (1.07, 1.74) | 0.012 |
| Q3 | 2.44 (2.15, 2.76) | < 0.001 | 1.79 (1.58, 2.03) | < 0.001 | 1.77 (1.40, 2.25) | < 0.001 |
| Q4 | 2.52 (2.23, 2.86) | < 0.001 | 1.86 (1.63, 2.11) | < 0.001 | 2.28 (1.78, 2.92) | < 0.001 |
| *P* for trend |  | < 0.001 |  | < 0.001 |  | < 0.001 |

Crude model: unadjusted.
Model I: adjusted for FPG, SBP, DBP, and family history of DM at baseline.

Model II: further adjusted for ALT, AST, LDL-C, HDL-C, TC, TG, BUN, Scr, smoking status, and drinking status at baseline.

**Supplementary Table 3. Relationship of CUN-BAE index with glycemic status conversion in an IFG population assessed by logistic regression in multiple models** **(the original dataset).**

| Variables | Crude model | | Model Ⅰ | | Model Ⅱ | |
| --- | --- | --- | --- | --- | --- | --- |
|  | OR (95%CI) | *P*-Value | OR (95%CI) | *P*-Value | OR (95%CI) | *P*-Value |
| IFG to normoglycemia | | |  |  |  |  |
| CUN-BAE index | 0.96 (0.96, 0.97) | < 0.001 | 0.97 (0.97, 0.98) | < 0.001 | 0.97 (0.96, 0.97) | < 0.001 |
| (CUN-BAE index quartiles) |  |  |  |  |  |  |
| Q1 | 1.00 (Reference) |  | 1.00 (Reference) |  | 1.00 (Reference) |  |
| Q2 | 0.60 (0.56, 0.64) | < 0.001 | 0.69 (0.64, 0.75) | < 0.001 | 0.63 (0.54, 0.73) | < 0.001 |
| Q3 | 0.57 (0.53, 0.61) | < 0.001 | 0.69 (0.64, 0.74) | < 0.001 | 0.69 (0.59, 0.80) | < 0.001 |
| Q4 | 0.53 (0.50, 0.57) | < 0.001 | 0.65 (0.60, 0.70) | < 0.001 | 0.53 (0.45, 0.63) | < 0.001 |
| *P* for trend |  | < 0.001 |  | < 0.001 |  | < 0.001 |
| IFG to DM | | |  |  |  |  |
| CUN-BAE index | 1.05 (1.04, 1.05) | < 0.001 | 1.03 (1.03, 1.04) | < 0.001 | 1.05 (1.04, 1.07) | < 0.001 |
| (CUN-BAE index quartiles) |  |  |  |  |  |  |
| Q1 | 1.00 (Reference) |  | 1.00 (Reference) |  | 1.00 (Reference) |  |
| Q2 | 2.45 (2.14, 2.79) | < 0.001 | 2.01 (1.75, 2.31) | < 0.001 | 1.83 (1.40, 2.40) | < 0.001 |
| Q3 | 2.91 (2.56, 3.32) | < 0.001 | 2.29 (1.99, 2.63) | < 0.001 | 2.19 (1.68, 2.85) | < 0.001 |
| Q4 | 2.75 (2.41, 3.13) | < 0.001 | 2.11 (1.83, 2.42) | < 0.001 | 2.71 (2.05, 3.58) | < 0.001 |
| *P* for trend |  | < 0.001 |  | < 0.001 |  | < 0.001 |

Crude model: unadjusted.
Model I: adjusted for FPG, SBP, DBP, and family history of DM at baseline.

Model II: further adjusted for ALT, AST, LDL-C, HDL-C, TC, TG, BUN, Scr, smoking status, and drinking status at baseline.

**Supplementary Table 4. Relationship of CUN-BAE index with glycemic status conversion in an IFG population assessed by Cox proportional-hazards regression in multiple models (included only participants with no missing covariates, n = 1964).**

| Variables | Crude model | | Model Ⅰ | | Model Ⅱ | |
| --- | --- | --- | --- | --- | --- | --- |
|  | HR (95%CI) | *P*-Value | HR (95%CI) | *P*-Value | HR (95%CI) | *P*-Value |
| IFG to normoglycemia | | |  |  |  |  |
| CUN-BAE index | 0.98 (0.96, 0.99) | < 0.001 | 0.98 (0.97, 0.99) | 0.004 | 0.98 (0.97, 0.99) | < 0.001 |
| (CUN-BAE index quartiles) |  |  |  |  |  |  |
| Q1 | 1.00 (Reference) |  | 1.00 (Reference) |  | 1.00 (Reference) |  |
| Q2 | 0.64 (0.54, 0.76) | < 0.001 | 0.70 (0.59, 0.83) | < 0.001 | 0.75 (0.63, 0.90) | 0.002 |
| Q3 | 0.59 (0.50, 0.71) | < 0.001 | 0.71 (0.60, 0.85) | < 0.001 | 0.72 (0.60, 0.87) | 0.001 |
| Q4 | 0.71 (0.60, 0.85) | < 0.001 | 0.83 (0.69, 0.99) | 0.037 | 0.75 (0.61, 0.92) | 0.006 |
| *P* for trend |  | < 0.001 |  | 0.023 |  | < 0.001 |
| IFG to DM | | |  |  |  |  |
| CUN-BAE index | 1.05 (1.03, 1.08) | < 0.001 | 1.05 (1.02, 1.07) | < 0.001 | 1.07 (1.04, 1.10) | < 0.001 |
| (CUN-BAE index quartiles) |  |  |  |  |  |  |
| Q1 | 1.00 (Reference) |  | 1.00 (Reference) |  | 1.00 (Reference) |  |
| Q2 | 3.01 (1.69, 5.34) | < 0.001 | 2.47 (1.38, 4.41) | 0.002 | 2.64 (1.47, 4.76) | 0.001 |
| Q3 | 3.50 (1.99, 6.16) | < 0.001 | 2.73 (1.55, 4.82) | 0.001 | 2.95 (1.66, 5.26) | < 0.001 |
| Q4 | 3.88 (2.19, 6.89) | < 0.001 | 3.30 (1.85, 5.89) | < 0.001 | 4.11 (2.23, 7.60) | < 0.001 |
| *P* for trend |  | < 0.001 |  | < 0.001 |  | < 0.001 |

Crude model: unadjusted.
Model I: adjusted for FPG, SBP, DBP, and family history of DM at baseline.

Model II: further adjusted for ALT, AST, LDL-C, HDL-C, TC, TG, BUN, Scr, smoking status, and drinking status at baseline.

**Supplementary Table 5. Relationship of CUN-BAE index with glycemic status conversion in an IFG population assessed by logistic regression in multiple models (included only participants with no missing covariates, n = 1964).**

| Variables | Crude model | | Model Ⅰ | | Model Ⅱ | |
| --- | --- | --- | --- | --- | --- | --- |
|  | OR (95%CI) | *P*-Value | OR (95%CI) | *P*-Value | OR (95%CI) | *P*-Value |
| IFG to normoglycemia | | |  |  |  |  |
| CUN-BAE index | 0.95 (0.94, 0.97) | < 0.001 | 0.96 (0.95, 0.98) | < 0.001 | 0.96 (0.95, 0.98) | < 0.001 |
| (CUN-BAE index quartiles) |  |  |  |  |  |  |
| Q1 | 1.00 (Reference) |  | 1.00 (Reference) |  | 1.00 (Reference) |  |
| Q2 | 0.50 (0.38, 0.64) | < 0.001 | 0.55 (0.42, 0.72) | < 0.001 | 0.59 (0.45, 0.78) | < 0.001 |
| Q3 | 0.44 (0.34, 0.57) | < 0.001 | 0.56 (0.42, 0.74) | < 0.001 | 0.61 (0.46, 0.81) | 0.001 |
| Q4 | 0.45 (0.35, 0.58) | < 0.001 | 0.55 (0.42, 0.73) | < 0.001 | 0.56 (0.41, 0.77) | < 0.001 |
| *P* for trend |  | < 0.001 |  | < 0.001 |  | < 0.001 |
| IFG to DM | | |  |  |  |  |
| CUN-BAE index | 1.05 (1.02, 1.07) | < 0.001 | 1.03 (1.00, 1.05) | 0.032 | 1.05 (1.01, 1.08) | 0.004 |
| (CUN-BAE index quartiles) |  |  |  |  |  |  |
| Q1 | 1.00 (Reference) |  | 1.00 (Reference) |  | 1.00 (Reference) |  |
| Q2 | 3.84 (2.13, 6.91) | < 0.001 | 3.32 (1.80, 6.15) | < 0.001 | 3.28 (1.75, 6.13) | < 0.001 |
| Q3 | 4.59 (2.57, 8.18) | < 0.001 | 3.49 (1.90, 6.40) | < 0.001 | 3.17 (1.70, 5.91) | < 0.001 |
| Q4 | 3.84 (2.13, 6.91) | < 0.001 | 2.79 (1.50, 5.16) | 0.001 | 3.32 (1.70, 6.46) | < 0.001 |
| *P* for trend |  | < 0.001 |  | 0.01 |  | 0.002 |

Crude model: unadjusted.
Model I: adjusted for FPG, SBP, DBP, and family history of DM at baseline.

Model II: further adjusted for ALT, AST, LDL-C, HDL-C, TC, TG, BUN, Scr, smoking status, and drinking status at baseline.

**Supplementary Table 6. Relationship of the CUN-BAE index with glycemic status conversion in an IFG population using the imputed dataset, assessed by Cox proportional-hazards regression with distinct covariate adjustments.**

| Variables | Crude model | | Model Ⅲ | |
| --- | --- | --- | --- | --- |
|  | HR (95%CI) | *P*-Value | HR (95%CI) | *P*-Value |
| IFG to normoglycemia | | |  |  |
| CUN-BAE index | 0.98 (0.98, 0.98) | < 0.001 | 0.99 (0.98, 0.99) | < 0.001 |
| (CUN-BAE index quartiles) |  |  |  |  |
| Q1 | 1.00 (Reference) |  | 1.00 (Reference) |  |
| Q2 | 0.70 (0.67, 0.73) | < 0.001 | 0.81 (0.77, 0.85) | < 0.001 |
| Q3 | 0.71 (0.68, 0.75) | < 0.001 | 0.82 (0.77, 0.86) | < 0.001 |
| Q4 | 0.72 (0.69, 0.76) | < 0.001 | 0.81 (0.77, 0.86) | < 0.001 |
| *P* for trend |  | < 0.001 |  | < 0.001 |
| IFG to DM | | |  |  |
| CUN-BAE index | 1.04 (1.04, 1.05) | < 0.001 | 1.04 (1.03, 1.04) | < 0.001 |
| (CUN-BAE index quartiles) |  |  |  |  |
| Q1 | 1.00 (Reference) |  | 1.00 (Reference) |  |
| Q2 | 1.98 (1.75, 2.25) | < 0.001 | 1.46 (1.29, 1.66) | < 0.001 |
| Q3 | 2.44 (2.15, 2.76) | < 0.001 | 1.75 (1.55, 1.99) | < 0.001 |
| Q4 | 2.52 (2.23, 2.86) | < 0.001 | 1.98 (1.73, 2.26) | < 0.001 |
| *P* for trend |  | < 0.001 |  | < 0.001 |

Crude model: unadjusted.
Model Ⅲ: adjusted for SBP, DBP, FPG, TC, TG, ALT, BUN, Scr and family history of DM at

baseline.

**Supplementary Table 7. Relationship of CUN-BAE index with glycemic status conversion in an IFG population defined by WHO criteria: results from Cox proportional-hazards regression in multiple models (n = 7193).**

| Variables | Crude model | | Model Ⅰ | | Model Ⅱ | |
| --- | --- | --- | --- | --- | --- | --- |
|  | HR (95%CI) | *P*-Value | HR (95%CI) | *P*-Value | HR (95%CI) | *P*-Value |
| IFG to normoglycemia | | |  |  |  |  |
| CUN-BAE index | 0.99 (0.98, 0.99) | < 0.001 | 0.99 (0.99, 0.99) | < 0.001 | 0.98 (0.98, 0.99) | < 0.001 |
| (CUN-BAE index quartiles) |  |  |  |  |  |  |
| Q1 | 1.00 (Reference) |  | 1.00 (Reference) |  | 1.00 (Reference) |  |
| Q2 | 0.67 (0.61, 0.73) | < 0.001 | 0.72 (0.65, 0.79) | < 0.001 | 0.75 (0.68, 0.82) | < 0.001 |
| Q3 | 0.73 (0.67, 0.81) | < 0.001 | 0.79 (0.72, 0.87) | < 0.001 | 0.79 (0.71, 0.87) | < 0.001 |
| Q4 | 0.80 (0.72, 0.87) | < 0.001 | 0.85 (0.77, 0.93) | 0.001 | 0.78 (0.70, 0.87) | < 0.001 |
| *P* for trend |  | < 0.001 |  | 0.003 |  | < 0.001 |
| IFG to DM | | |  |  |  |  |
| CUN-BAE | 1.02 (1.01, 1.03) | < 0.001 | 1.02 (1.01, 1.03) | < 0.001 | 1.03 (1.02, 1.04) | < 0.001 |
| (CUN-BAE index quartiles) |  |  |  |  |  |  |
| Q1 | 1.00 (Reference) |  | 1.00 (Reference) |  | 1.00 (Reference) |  |
| Q2 | 1.34 (1.17, 1.53) | < 0.001 | 1.26 (1.10, 1.44) | 0.001 | 1.2 (1.05, 1.38) | 0.008 |
| Q3 | 1.41 (1.23, 1.61) | < 0.001 | 1.32 (1.15, 1.51) | < 0.001 | 1.3 (1.13, 1.50) | < 0.001 |
| Q4 | 1.44 (1.26, 1.65) | < 0.001 | 1.39 (1.21, 1.59) | < 0.001 | 1.59 (1.36, 1.85) | < 0.001 |
| *P* for trend |  | < 0.001 |  | < 0.001 |  | < 0.001 |

Crude model: unadjusted.
Model I: adjusted for FPG, SBP, DBP, and family history of DM at baseline.

Model II: further adjusted for ALT, AST, LDL-C, HDL-C, TC, TG, BUN, Scr, smoking status, and drinking status at baseline.

**Supplementary Table 8.**  **Relationship of CUN-BAE index with glycemic status conversion in an IFG population defined by WHO criteria: results from logistic regression in multiple models (n = 7193).**

| Variables | Crude model | | Model Ⅰ | | Model Ⅱ | |
| --- | --- | --- | --- | --- | --- | --- |
|  | OR (95%CI) | *P*-Value | OR (95%CI) | *P*-Value | OR (95%CI) | *P*-Value |
| IFG to normoglycemia | | |  |  |  |  |
| CUN-BAE index | 0.98 (0.97, 0.98) | < 0.001 | 0.98 (0.97, 0.99) | < 0.001 | 0.97 (0.96, 0.98) | < 0.001 |
| (CUN-BAE index quartiles) |  |  |  |  |  |  |
| Q1 | 1.00 (Reference) |  | 1.00 (Reference) |  | 1.00 (Reference) |  |
| Q2 | 0.57 (0.50, 0.65) | < 0.001 | 0.62 (0.54, 0.71) | < 0.001 | 0.65 (0.57, 0.75) | < 0.001 |
| Q3 | 0.62 (0.55, 0.71) | < 0.001 | 0.69 (0.60, 0.80) | < 0.001 | 0.7 (0.61, 0.80) | < 0.001 |
| Q4 | 0.66 (0.58, 0.76) | < 0.001 | 0.73 (0.63, 0.83) | < 0.001 | 0.64 (0.55, 0.75) | < 0.001 |
| *P* for trend |  | < 0.001 |  | < 0.001 |  | < 0.001 |
| IFG to DM | | |  |  |  |  |
| CUN-BAE index | 1.03 (1.02, 1.03) | < 0.001 | 1.02 (1.01, 1.03) | < 0.001 | 1.04 (1.03, 1.05) | < 0.001 |
| (CUN-BAE index quartiles) |  |  |  |  |  |  |
| Q1 | 1.00 (Reference) |  | 1.00 (Reference) |  | 1.00 (Reference) |  |
| Q2 | 1.79 (1.53, 2.09) | < 0.001 | 1.67 (1.43, 1.96) | < 0.001 | 1.55 (1.32, 1.83) | < 0.001 |
| Q3 | 1.78 (1.52, 2.07) | < 0.001 | 1.63 (1.39, 1.91) | < 0.001 | 1.62 (1.37, 1.91) | < 0.001 |
| Q4 | 1.64 (1.41, 1.92) | < 0.001 | 1.52 (1.29, 1.79) | < 0.001 | 1.87 (1.55, 2.25) | < 0.001 |
| *P* for trend |  | < 0.001 |  | < 0.001 |  | < 0.001 |

Crude model: unadjusted.
Model I: adjusted for FPG, SBP, DBP, and family history of DM at baseline.

Model II: further adjusted for ALT, AST, LDL-C, HDL-C, TC, TG, BUN, Scr, smoking status, and drinking status at baseline.

**Supplementary Table 9. Association of the CUN-BAE index with bidirectional glycemic transitions in an IFG population assessed by competing risk regression (Fine-Gray model, n = 26247).**

| Variables | Crude model | | Model Ⅰ | | Model Ⅱ | |
| --- | --- | --- | --- | --- | --- | --- |
|  | SHR (95%CI) | *P*-Value | SHR (95%CI) | *P*-Value | SHR (95%CI) | *P*-Value |
| IFG to normoglycemia | | |  |  |  |  |
| CUN-BAE index | 0.97 (0.97, 0.98) | < 0.001 | 0.99 (0.98, 0.99) | < 0.001 | 0.98 (0.98, 0.99) | < 0.001 |
| (CUN-BAE index quartiles) |  |  |  |  |  |  |
| Q1 | 1.00 (Reference) |  | 1.00 (Reference) |  | 1.00 (Reference) |  |
| Q2 | 0.67 (0.64, 0.70) | < 0.001 | 0.77 (0.74, 0.81) | < 0.001 | 0.81 (0.78, 0.85) | < 0.001 |
| Q3 | 0.66 (0.63, 0.69) | < 0.001 | 0.78 (0.75, 0.82) | < 0.001 | 0.80 (0.76, 0.84) | < 0.001 |
| Q4 | 0.66 (0.63, 0.69) | < 0.001 | 0.80 (0.76, 0.84) | < 0.001 | 0.77 (0.73, 0.81) | < 0.001 |
| *P* for trend |  | < 0.001 |  | < 0.001 |  | < 0.001 |
| IFG to DM | | |  |  |  |  |
| CUN-BAE | 1.05 (1.04, 1.05) | < 0.001 | 1.03 (1.03, 1.04) | < 0.001 | 1.04 (1.03, 1.05) | < 0.001 |
| (CUN-BAE index quartiles) |  |  |  |  |  |  |
| Q1 | 1.00 (Reference) |  | 1.00 (Reference) |  | 1.00 (Reference) |  |
| Q2 | 2.42 (2.14, 2.74) | < 0.001 | 1.79 (1.59, 2.02) | < 0.001 | 1.71 (1.51, 1.93) | < 0.001 |
| Q3 | 2.91 (2.58, 3.28) | < 0.001 | 2.08 (1.84, 2.34) | < 0.001 | 2.02 (1.79, 2.27) | < 0.001 |
| Q4 | 2.88 (2.56, 3.25) | < 0.001 | 2.03 (1.80, 2.29) | < 0.001 | 2.18 (1.91, 2.48) | < 0.001 |
| *P* for trend |  | < 0.001 |  | < 0.001 |  | < 0.001 |

Crude model: unadjusted.
Model I: adjusted for FPG, SBP, DBP, and family history of DM at baseline.

Model II: further adjusted for ALT, AST, LDL-C, HDL-C, TC, TG, BUN, Scr, smoking status, and drinking status at baseline.
